# 5-Hydroxymethylcytosine-mediated active demethylation is required for mammalian neuronal differentiation and function

**DOI:** 10.7554/eLife.66973

**Published:** 2021-12-17

**Authors:** Elitsa Stoyanova, Michael Riad, Anjana Rao, Nathaniel Heintz

**Affiliations:** 1 Laboratory of Molecular Biology, Howard Hughes Medical Institute, The Rockefeller University New York United States; 2 Sanford Consortium for Regenerative Medicine La Jolla United States; 3 La Jolla Institute for Allergy and Immunology La Jolla United States; 4 Department of Pharmacology, University of California San Diego La Jolla United States; Duke University School of Medicine United States; Harvard University United States

**Keywords:** 5-hydroxymethylcytosine, DNA demethylation, TET proteins, Mouse

## Abstract

Although high levels of 5-hydroxymethylcytosine (5hmC) accumulate in mammalian neurons, our knowledge of its roles in terminal differentiation or as an intermediate in active DNA demethylation is incomplete. We report high-resolution mapping of DNA methylation and hydroxymethylation, chromatin accessibility, and histone marks in developing postmitotic Purkinje cells (PCs) in *Mus musculus*. Our data reveal new relationships between PC transcriptional and epigenetic programs, and identify a class of genes that lose both 5-methylcytosine (5mC) and 5hmC during terminal differentiation. Deletion of the 5hmC writers Tet1, Tet2, and Tet3 from postmitotic PCs prevents loss of 5mC and 5hmC in regulatory domains and gene bodies, and hinders transcriptional and epigenetic developmental transitions. Our data demonstrate that Tet-mediated active DNA demethylation occurs in vivo, and that acquisition of the precise molecular properties of adult PCs require continued oxidation of 5mC to 5hmC during the final phases of differentiation.

## eLife digest

At birth, the mammalian brain contains tens of billions of neurons. Although the number does not increase much as the animal grows, there are many dramatic changes to their size and structure. These changes allow the neurons to communicate with one another, develop into networks, and learn the tasks of the adult brain. One way that these changes occur is by the accumulation of chemical marks on each neuron’s DNA that help dictate which genes switch on, and which turn off.

One of the most common ways that DNA can be marked is through the addition of a chemical group called a methyl group to one of the four DNA bases, cytosine. This process is called methylation. When methylation occurs, cytosine becomes 5-methylcytosine, or 5mC for short.

In 2009, researchers found another modification present in the DNA in the brain: 5-hydroxymethylcytosine, or 5hmC. This modification appears when a group of proteins called the Tet hydroxylases turn 5mC into 5hmC. Converting 5mC to 5hmC normally helps cells remove marks on their DNA before they divide and expand. This is important because the newly generated cells need to be able to accumulate their own methylation marks to perform their roles properly. However, neurons in the brain accumulate 5hmC after birth, when the cells are no longer dividing, indicating that 5hmC may be required for the neurons to mature.

Stoyanova et al. set out to determine whether mouse neurons need 5hmC to get their adult characteristics by tracking the chemical changes that occur in DNA from birth to adulthood. Some of the mice they tested produced 5hmC normally, while others lacked the genes necessary to make the Tet proteins in a specific class of neurons, preventing them from converting 5mC to 5hmC as they differentiate. The results reveal that neurons do not mature properly if 5hmC is not produced continuously following the first week of life. This is because neurons need to have the right genes switched on and off to differentiate correctly, and this only happens when 5hmC accumulates in some genes, while 5hmC and 5mC are removed from others. The data highlight the role of the Tet proteins, which convert 5mC into 5hmC, in preparing the marks for removal and demonstrate that active removal of these marks is essential for neuronal differentiation.

Given the role of 5hmC in the development of neurons, it is possible that problems in this system could contribute to brain disorders. Further studies aimed at understanding how cells control 5hmC levels could lead to new ways to improve brain health. Research has also shown that if dividing cells lose the ability to make 5hmC, they can become cancerous. Future work could explain more about how and why this happens.

## Introduction

Development of the mammalian brain requires generation of hundreds of millions of neuronal progenitors that differentiate into distinct cell types with refined functional properties. Although morphological and physiological maturation of most neurons occurs between mid-gestation and a few months or years after birth, the vast majority of CNS neurons must maintain a stable differentiated state for the life of the organism and remain sufficiently plastic to participate in novel behaviors. Studies of signaling molecules and transcriptional programs have identified many mechanisms that orchestrate critical steps in neurogenesis, cell type diversification, neuronal migration, axonal pathfinding, and differentiation. Although it has been established that epigenetic regulatory mechanisms are critical for proper development of all cell types, our knowledge of the precise roles of these mechanisms in neuronal differentiation, function, and vitality remains rudimentary.

5-Hydroxymethylcytosine (5hmC) is produced from 5-methylcytosine (5mC) by the Ten-eleven translocation dioxygenases (Tet1, Tet2, Tet3) (Iyer et al., 2009; Tahiliani et al., 2009). It is present at approximately 10-fold higher levels in neurons than peripheral cell types (Globisch et al., 2010; Kriaucionis and Heintz, 2009), and its distribution across the genome of adult neurons is cell specific and correlated with active gene expression (Mellén et al., 2017; Mellén et al., 2012; Szulwach et al., 2011). The initial discovery that 5hmC accumulates within active gene bodies, coupled with the discovery that MeCP2 can bind probes containing 5hmC with high affinity, led to the proposal that MeCP2 binding within active genes facilitates their expression (Mellén et al., 2012). Further studies demonstrating that MeCP2 binds 5hmC at high affinity in non-CG dinucleotides but does not bind to 5hmCG overturned this model (Ayata, 2013; Gabel et al., 2015; Mellén et al., 2017) by revealing that accumulation of 5hmCG and the depletion of 5hmCH in gene bodies is correlated with less MeCP2 binding and increased expression (Ayata, 2013; Gabel et al., 2015; Mellén et al., 2017). Imaging of the binding and diffusion of single MeCP2 molecules in living neurons lacking Dnmt3a or Tet1, Tet2, and Tet3 demonstrate that its binding is exquisitely sensitive to the levels of both 5mC and 5hmC (Piccolo et al., 2019). Given these data and the sensitivity of neuronal function to MeCP2 gene dosage (Chahrour et al., 2008; Nan and Bird, 2001), relief of the repressive functions of MeCP2 through Tet-mediated conversion of high-affinity 5mCG-binding sites to low-affinity 5hmCG sites, which we have referred to as functional demethylation (Mellén et al., 2017), provides an important mechanism for modulation of chromatin structure and transcription.

In dividing cells, 5hmC serves as an intermediate in DNA demethylation because maintenance DNA methyltransferases do not recognize hemi-hydroxymethylated cytosines in order to reestablish methylation (Wu and Zhang, 2017). Consequently, 5hmC is lost passively and replaced by C due to replicative dilution. Loss of function studies in mouse embryonic stem cells (ESCs) (Dawlaty et al., 2013) and lymphocyte lineages Lio and Rao, 2019 have demonstrated that the role of Tet-mediated replicative DNA demethylation is to provide full accessibility to regulatory regions necessary for expression of genes required for differentiation. For example, at the activation-induced deaminase (AID) locus in B cells, Tet activity is required for demethylation and activation of enhancer regions that modulate AID expression to enable class switch recombination (Lio et al., 2019). Tet-mediated replication-dependent passive demethylation is thought to be common in many dividing cell types, including progenitor cells in the developing nervous system (MacArthur and Dawlaty, 2021).

Most neurons exit the cell cycle during mid-gestation and remain relatively simple and undifferentiated until birth. Following parturition, they initiate an elaborate program of differentiation that includes dramatic increases in size, morphological complexity, and connectivity. Since these neurons are postmitotic and remain so throughout life, removal of the repressive effects of 5mC by passive demethylation cannot occur. However, in addition to functional demethylation and passive demethylation, a third pathway for DNA demethylation, often referred to as active demethylation, has been proposed based on the finding that 5hmC can be further oxidized by Tet proteins to produce 5-formylcytosine (5fC) and 5-carboxylcytosine (5caC). Removal of 5fC and 5caC by thymine DNA glycosylase (TDG)-dependent base excision repair (BER) provides another mechanism for 5hmC-mediated DNA demethylation (He et al., 2011). Clear evidence that this pathway can operate in cultured cells has been presented, and detailed biochemical studies have delineated the mechanisms operating in TDG-BER DNA demethylation (Weber et al., 2016). Active DNA demethylation is ideally suited for remodeling DNA methylation in postmitotic neurons.

Evidence that continued accumulation of 5hmC is required in differentiating neurons and that it can participate in active demethylation in vivo is beginning to emerge. In studies of cerebellar granule cell development in ESC-derived GC cultures, primary GC cultures, and slice preparations, manipulations of Tet activity and 5hmC levels provide strong evidence that 5hmC is required for expression of axon guidance and ion channel genes, and that proper development of the GC dendritic arbor requires 5hmC (Zhu et al., 2016). Demethylation of 5hmC-GFP transfected DNA fragments retrieved after several days in culture display reduced 5hmC levels at several sites as assessed by bisulfite sequencing (BSSeq) in HEK 293 cells and primary hippocampal neurons (Guo et al., 2011). Changes in methylation levels at GGCC sites in the genome of hippocampal dentate gyrus neurons in response to electroconvulsive shock have been documented using the methylation-sensitive cut counting method that employs the methylation or hydroxymethylation-sensitive restriction enzyme HpaII and its methylation-insensitive isoschizomer Msp1 (Guo et al., 2011). Global measurements of 5mC and 5hmC by mass spectroscopy in the hippocampus have shown that both decrease in response to the induction of seizure (Kaas et al., 2013). And in germline Tet1 knockout mice, analysis of the Npas4 and c-Fos promoters has shown that Tet1 is required for their proper regulation in both the cerebral cortex and hippocampus (Rudenko et al., 2013). Despite these observations, our knowledge of the roles of continued 5hmC accumulation in active DNA demethylation and postmitotic differentiation remains rudimentary.

We report here single nucleotide resolution studies of DNA methylation and hydroxymethylation, transcription, chromatin accessibility, and H3K4me3 and H3K27me3 histone marks in postmitotic, differentiating Purkinje cells (PCs). As PCs transition from relatively small, multipolar immature cells to fully elaborated large neurons with complex dendritic arbors and hundreds of thousands of synapses (McKay and Turner, 2005), 5hmC continues to accumulate, and DNA methylation and hydroxymethylation are reconfigured as epigenetic and transcriptional programs progress. Our data confirm previous studies of the relationships between transcription, DNA methylation, DNA hydroxymethylation, and chromatin organization (Lister et al., 2013; Mellén et al., 2017; Tsagaratou et al., 2017; Tsagaratou et al., 2014), and they reveal several novel modes of transcriptional activation and repression. Notably, we identify a class of developmentally induced PC-specific genes that are highly expressed and lose both 5mC and 5hmC in the final stages of PC differentiation.

We report also studies of newly generated *Tet1*, *Tet2*, *Tet3* PC-specific triple knockout (Pcp2TetTKO) mouse lines in which recombination is activated in the first postnatal week. These data demonstrate that postmitotic transcriptional and epigenetic maturation in PCs, including transcription of many ion channels and active demethylation of late expressed genes, requires continued oxidation of 5mC to 5hmC. Taken together, our data demonstrate that active demethylation occurs in select genes in postmitotic neurons, and that 5hmC plays an essential role in refining the transcriptional and epigenetic status of PCs during the final stages of differentiation.

## Results

**Figure 1.**
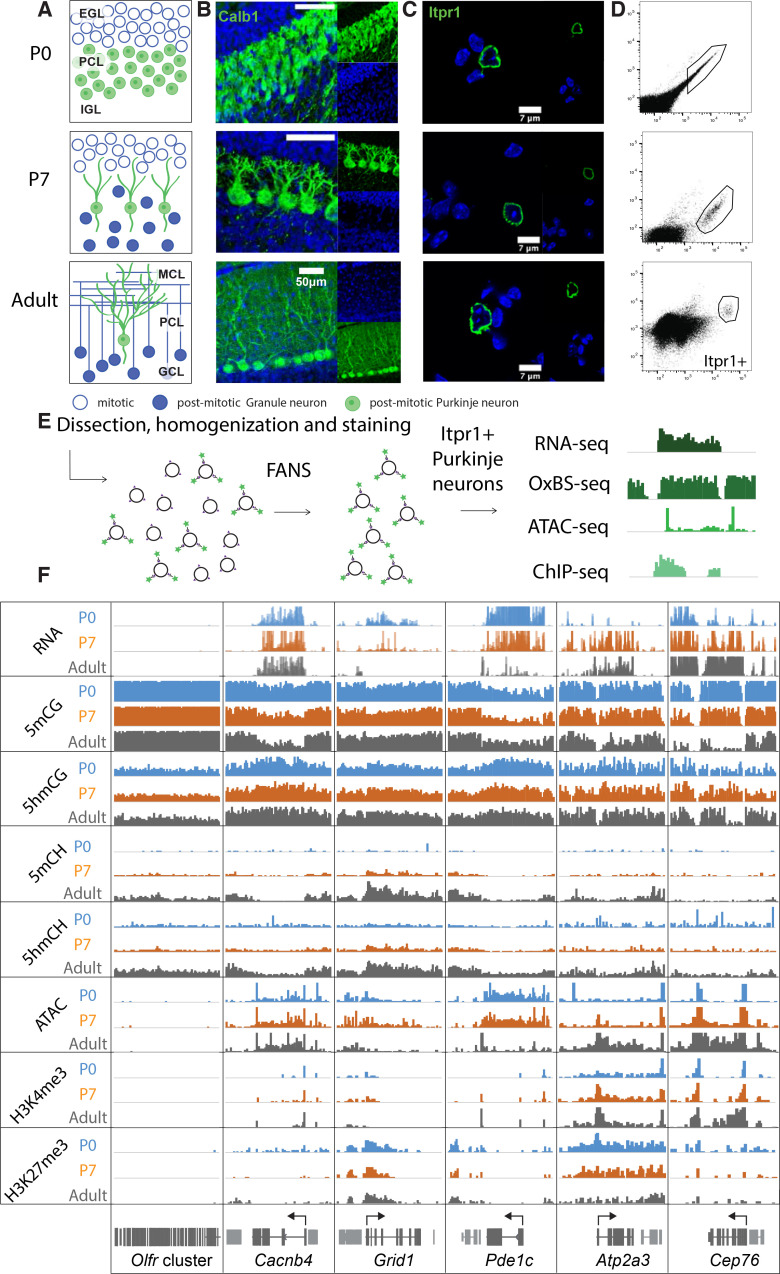
Chromatin landscape in differentiating Purkinje neurons. (**A**) Schematic of Purkinje cell (PC) differentiation and growth at P0, P7, and adult (approximately 8-week-old) timepoints. EGL – external granule layer, PCL – Purkinje cell layer, IGL – internal granule layer, MCL – molecular cell layer, GCL – granule cell layer. (**B**) Immunofluorescence staining with Calb1 (green) of PCs in murine cerebella at P0, P7, and adult timepoints. (**C**) Example of PC nuclei stained with Itpr1 (green) post-dissociation and pre-sorting, counterstained with DAPI. (**D**) Representative plots of fluorescence-activated nuclear sorting of PCs at P0, P7, and adult timepoints with Itpr1. (**E**) Workflow schematic of nuclei isolation, antibody staining with anti-Itpr1, fluorescence-activated sorting, and downstream sequencing applications. (**D**) Integrated genome viewer (IGV) representation of example regions of differentially regulated genes (*Olfr* cluster – always silent, *Cacnb4* – always expressed, *Grid1* and *Pde1c* – developmentally down-regulated, *Atp2a3* and *Cep76* – developmentally up-regulated). Top tracks show RNA expression in RPKM (reads per kilobase per million mapped reads), mCG tracks show methylation level in CG context from 0 to 0.8, hmCG tracks show hydroxymethylation level in CG context from 0 to 0.45, mCH and hmCH show methylation and hydroxymethylation level in CH context (H = A, C, or T) from 0 to 0.04. ATAC tracks show ATACSeq read density in RPKM from 0 to 10. H3K4me3 tracks show input normalized enrichment in RPKM from 0 to 5. H3K27me3 tracks show input normalized enrichment in RPKM from 0 to 3.

In mice, PC progenitors complete their final cell cycle between e10.5 and e13.5 within the ventricular zone of the developing cerebellar anlage (Leto et al., 2016). As they exit cell cycle and commit to a PC fate, they express the transcription factors Lhx1/Lhx5 (Chizhikov et al., 2006; Morales and Hatten, 2006). To characterize 5hmC accumulation, transcription and chromatin organization during their postmitotic development, we chose to analyze PCs at the start of their accelerated differentiation (P0), during their rapid phase of morphological development (P7) and as fully mature, differentiated neurons (adult) (Figure 1A–B). We employed fluorescence-activated nuclear sorting (FANS) to purify Itpr1-positive PC nuclei in two biological replicates (Figure 1C–E, Figure S1A) (Xu et al., 2018). Since PCs are extremely rare and difficult to isolate, we optimized low input protocols for genomic profiling. We used ~20,000 nuclei for transcriptional profiling, ~200,000 nuclei for BSSeq and oxidative bisulfite sequencing (OxBSSeq), 25,000 nuclei for the assay for transposase-accessible chromatin sequencing (ATACSeq), and 25,000 nuclei for H3K27me3 and H3K4me3 chromatin immunoprecipitation sequencing (ChIPSeq) (Figure 1E–F). Analysis of biological replicates for each of the assays revealed that the sorted nuclei expressed markers of Purkinje neurons, and did not express genes marking the two most abundant cerebellar cell types, granule cells and glia (Figure S1B). The Pearson correlation coefficients between each replicate at all timepoints and techniques (RNASeq, OxBSSeq, ATACSeq, and ChIPSeq) were high (over 0.9), establishing that our data are reproducible and of very high quality (Figure 1—figure supplement 1C-F). Bisulfite conversion and oxidation rates for the OxBSSeq datasets were within the expected range (Figure 1—figure supplement 1G).

**Figure 1—figure supplement 1.**
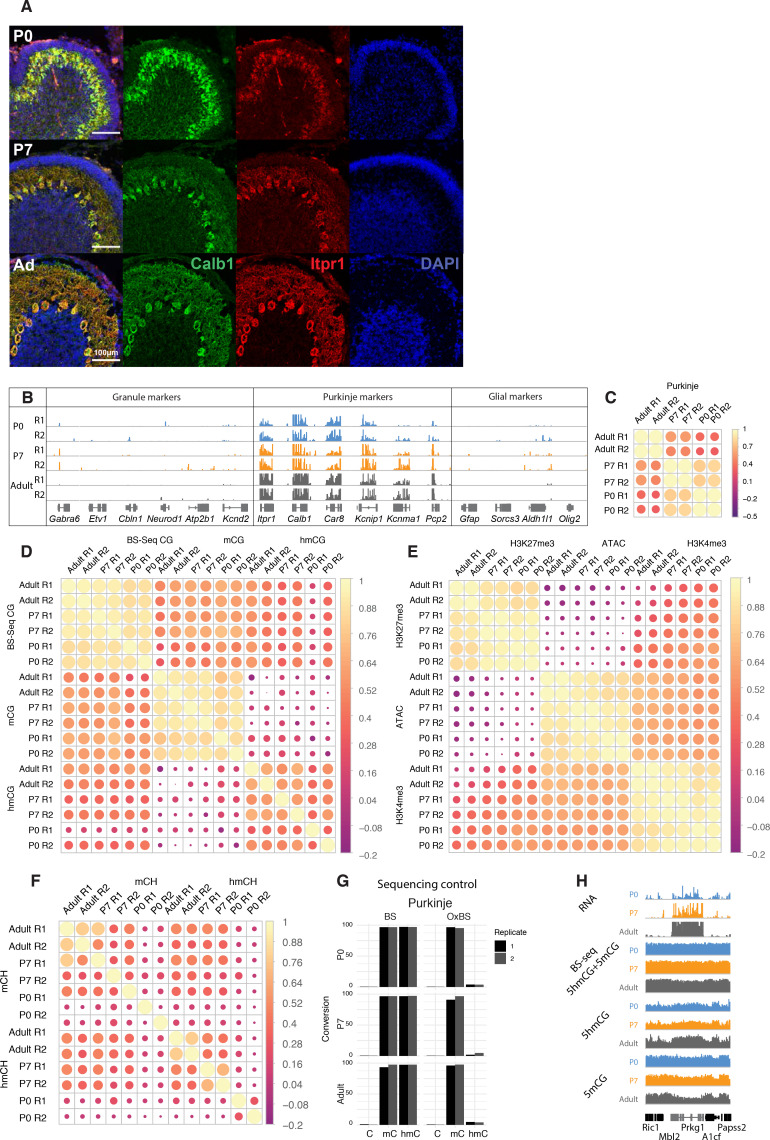
Quality control of sequencing datasets. (**A**) Immunofluorescence staining of Purkinje cells at P0, P7, and adult timepoints with Calb1 (green) and Itpr1 (red). (**B**) Integrated genome viewer (IGV) representation of Purkinje-specific markers (*Itpr1, Calb1, Car8, Kcnip1, Kcnma1, Pcp2*) enrichment and depletion of granule (*Gabra6, Etv1, Cbln1, Neurod1, Atp2b1, Kcnd2*) and glial (*Gfap, Sorcs3, Aldh1l1, Olig2*) markers. (**C**) Pearson correlation of RNASeq datasets. (**D**) Pearson correlation of gene body accumulation of 5hmCG + 5 mCG (bisulfite sequencing [BSSeq]), 5hmCG, and 5mCG (derived from maximum likelihood estimator model of BSSeq and oxidative bisulfite sequencing [OxBSSeq]). (**E**) Pearson correlation of assay for transposase-accessible chromatin sequencing (ATACSeq), H3K4me3, and H3K27me3 chromatin immunoprecipitation sequencing (ChIPSeq) dataset enrichment ±5 kb around the gene promoters. (**F**) Pearson correlation of gene body accumulation of 5hmCH and 5mCH (derived from maximum likelihood estimator model of BSSeq and OxBSeq). (**G**) Bisulfite conversion and oxidation efficiency in BSSeq and OxBSSeq datasets. (**H**) IGV representation of BSSeq (showing 5hmCG + 5 mCG signal together) and OxBSSeq (showing 5hmCG + 5 mCG signal separately) at the three developmental timepoints.

It is important to note that we chose to focus on OxBSSeq (Booth et al., 2013) as a definitive technology for analysis of DNA methylation because, in contrast to BSSeq alone, it allows us to assess the contributions of both 5mC and 5hmC to neuronal development. Given the distinct functions of these two modifications, analysis of their separate contributions to epigenetic regulation of the genome is more informative than BSSeq alone. For example, in many genes, 5mCG is depleted and 5hmCG accumulates in the gene body as its expression increases between P0 and adult. This is clearly evident for the gene encoding the cyclic GMP-dependent protein kinase (*Prkg1*), whose expression in PCs has been shown to be essential for long-term depression (Feil et al., 2003). In this case, the OxBSSeq data reveal a transition from 5mCG to 5hmCG that cannot be detected in the BSSeq data as the gene is activated between P0 and adult (Figure 1—figure supplement 1H). These data illustrate that a precise evaluation of DNA methylation status in relation to other programs unfolding during development for this gene and many others is advanced significantly by inclusion of OxBSSeq data.

### Transcriptional programs altered during PC differentiation

PC progenitors complete their final divisions in the cerebellar primordium between e11 and e13 (Butts et al., 2014) and remain as a multilayered, simple migrating cell population until birth (P0). In the first postnatal week, they organize into a monolayer as the cerebellum enlarges and begin the transition from a multipolar primitive neuronal morphology to one of the largest neurons in the brain with a characteristic, highly elaborate planar dendritic arbor. As they begin the second postnatal week (P7), PCs undergo an important developmental transition that includes refinement of their climbing fiber input, formation of many thousands of parallel fiber synapses, and myelination of their axons (Leto et al., 2016; McKay and Turner, 2005). The tremendous increase in synaptogenesis and connectivity continues until PCs attain their mature morphology and functions at 3–4 weeks of age. As these programs unfold, there is a global decrease of 5mCG and global increase of 5hmCG (Figure 2—figure supplement 1A).

**Figure 2.**
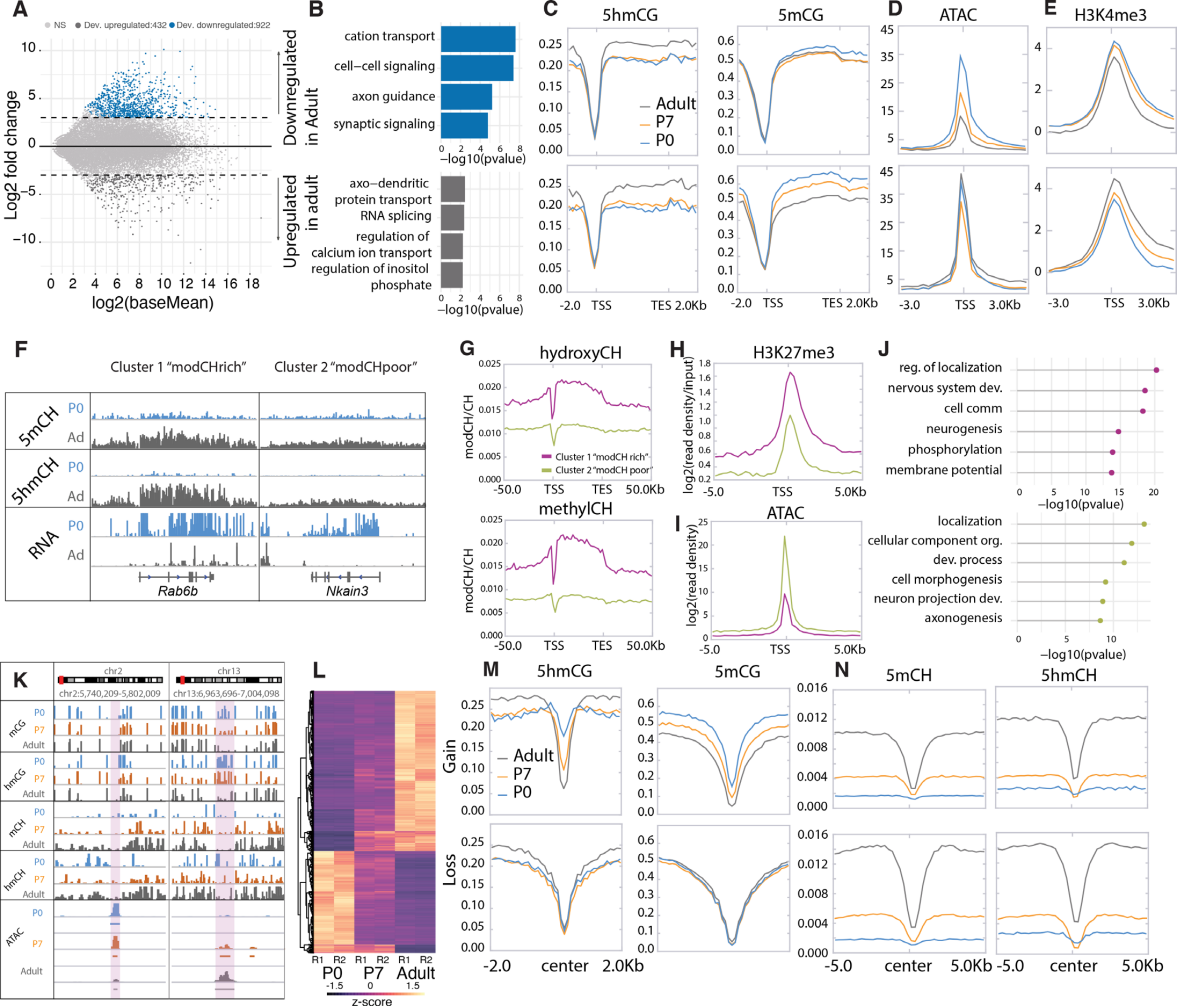
Cytosine modification dynamics in in relation to differential expression and accessibility. (**A**) MA plot representing statistically significant (p < 0.01, log2(fold change) > 2) differential gene expression between P0 (blue) and adult (dark gray). Light gray dots represent genes that are not statistically significant. (**B**) Gene ontology analysis of the developmentally up- and down-regulated genes. (**C–E**) Metagene plots representing the mean value of 5hmCG/CG, 5mCG/CG (**C**), assay for transposase-accessible chromatin sequencing (ATACSeq) read density (**D**) and H3K4me3 accumulation (**E**) over the gene bodies and promoters of developmentally up- and down-regulated genes over the three timepoints (P0 – blue, P7 – orange, and adult – dark gray). (**F**) Genome browser representation of the two classes of developmentally down-regulated genes with differential accumulation of CpH modifications. (**G–I**) Metagene plots representing the mean value of 5hmCH/CH, 5mCH/CH (**G**), ATACSeq read density (**H**), and H3K27me3 accumulation (**I**) over the gene bodies and promoters of developmentally down-regulated genes divided into two clusters by k-means clustering analysis (green for ‘modCHpoor’, purple for ‘modCHrich’). (**J**) Gene ontology analysis of the two clusters. (**K**) Genome browser representation of two regions with differential accessibility. (**L**) Heatmap representing the differentially accessible regions between P0 and adult Purkinje cells (PCs) (p < 0.01, log2(fold change) > 4). (**M–N**) Metagene plots representing the mean values of 5hmCG/CG and 5mCG/CG (**M**) and 5hmCH/CH and 5mCH/CH (**N**) over the centers and flanking regions of peaks that gained or lost accessibility relative to adult PCs.

To identify genes whose transcription is stable and those that are dynamically regulated during PC differentiation, we conducted differential expression analysis between P0 and adult Purkinje neurons, filtering for significance of p < 0.01 and log2 fold change of >2 in either direction (Figure 2A–B, Supplementary file 1). Using these criteria, there were 922 developmentally repressed genes (down-regulated in adult) enriched in categories involved in cell signaling and axon guidance pathways (e.g. *Grid1*, *Pde1c*; Figure 1F). We identified 432 genes with increased expression as PCs differentiate encoding proteins known to be important for the mature functions of PCs including calcium ion buffering and transport, regulation of the inositol triphosphate signaling pathway, and RNA splicing. As expected, similar analysis comparing P0 and P7 or P7 and adult data revealed genes overlapping with those identified in the overall P0 and adult comparative data (Figure 2—figure supplement 1B-D), although additional categories of RNA metabolism, synaptic signaling, and ion transport are enriched in the P7 to adult analysis (Figure 2—figure supplement 1C). As anticipated, the P7 profiles we have included in our analysis have been essential in assessing both the developmental course of epigenetic events studied here and the consequences of loss of 5hmC in PCs (see below).

**Figure 2—figure supplement 1.**
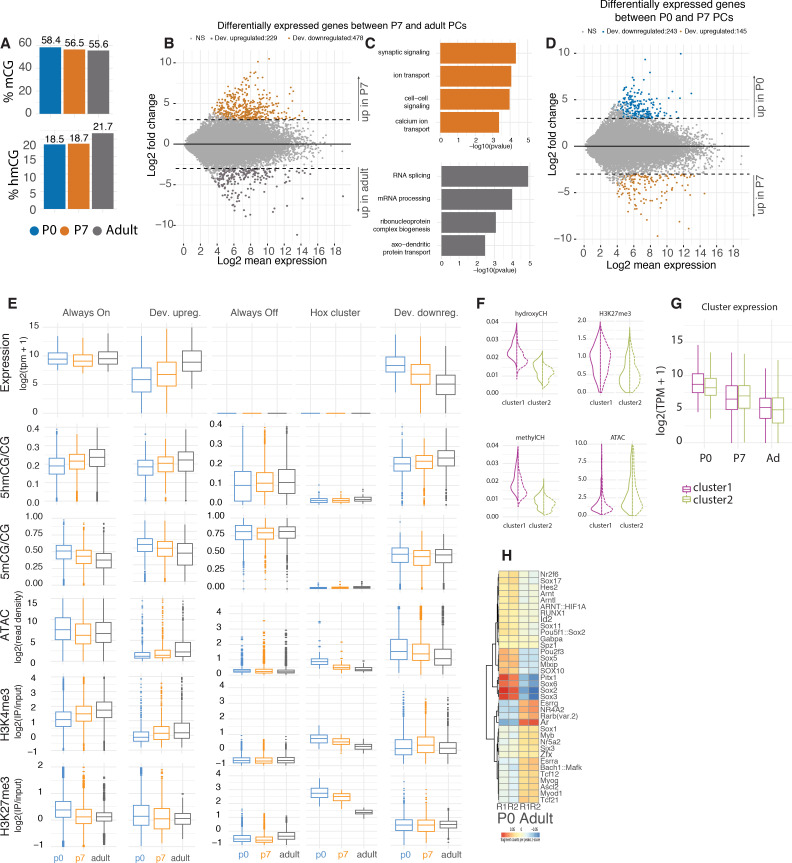
Gene expression and chromatin dynamics during Purkinje cell (PC) development. (**A**). Genome-wide quantification of the levels of 5hmCG and 5mCG at each timepoint. (**B**) MA plot representing differential gene expression between P7 and adult. Orange shaded dots represent genes enriched in P7 and gray dots represent genes enriched in adult, with absolute log2 fold change of 2 and p-adj value of 0.05. (**C**) Gene ontology (GO) analysis of differentially expressed genes between P7 and adult. (**D**) MA plot representing differential gene expression between P0 and P7 timepoints. Blue shaded dots represent genes enriched in P0 and orange dots represent genes enriched in P7, with absolute log2 fold change of 2 and p-adj value of 0.05. (**E**) Quantification of expression, chromatin accessibility, 5mCG, 5hmCG, H3K4me3, and H3K27me3. (**F**) Violin plots of the quantifications of 5hmCH, 5mCH, ATAC, and H3K27me3 density over the two clusters of repressed genes. (**G**) Expression levels log2(TPM + 1) of the genes in each cluster. (**H**) Differentially abundant motifs of transcription factors in chromatin accessibility data between P0 and adult PCs.

### Constitutively expressed genes are epigenetically stable

Despite the many genes that are dynamically regulated during these developmental transitions, the majority of genes in PCs are either silent or constitutively expressed. Approximately half of the genes in the mouse genome (e.g. genes encoding olfactory receptors (Figure 1F), immunoglobulins, hemoglobin subunits, etc.) are never expressed in PCs and remain heavily methylated and inaccessible. As previously reported for other neurons (Mo et al., 2015), there is a second class of silent genes in PCs that are enriched in transcription factors expressed in very early embryos (Hox clusters, other homeobox genes, etc.). These are completely demethylated and enriched for the H3K27me3 histone mark, indicating that they are repressed by the polycomb repressive complex (Li et al., 2018).

Actively transcribed genes whose levels vary little during PC differentiation, for example the calcium channel auxiliary subunit *Cacnb4* (Figure 1F), are also epigenetically stable. This is a large (~3000) and diverse class of genes that carry activating epigenetic marks that have been previously characterized: low levels of 5mCG, 5mCH, and 5hmCH; elevated levels of 5hmCG; they are ATAC accessible; and their promoters carry the activating histone mark H3K4me3. As expected, the levels of these marks vary widely between genes and generally reflect the level of expression. As PCs mature, the conversion of 5mC to 5hmC continues to increase within the gene bodies of the most active constitutively expressed genes without a strong impact on expression (Figure 2—figure supplement 1E).

### Epigenetic signatures reveal two classes of developmentally repressed genes in PC

To identify mechanisms associated with developmental repression, we analyzed features previously associated negatively with transcription in the 922 genes whose expression decreases during PC differentiation. A consistent finding is that in most genes decreased expression is associated with a loss of promoter accessibility as assayed by assay for transposase-accessible chromatin sequencing (ATACSeq) (Figure 2D and K–L, Figure 2—figure supplement 1E). These changes are not correlated with changes in DNA methylation or H3K27me3 occupancy (Figure 2C, Figure 2—figure supplement 1E). Furthermore, 5hmCG accumulated over the genes bodies early in development is stable or continues to increase (Figure 2C), suggesting that the presence of 5hmCG is not sufficient to maintain transcription. Interestingly, analysis of the repressive marks 5mCH and 5hmCH reveals at least two distinctly recognizable epigenetic patterns in genes that become repressed in PC development (Figure 2F–J, Figure 2—figure supplement 1F-G). Repressed genes that accumulate 5mCH and 5hmCH over their gene bodies (referred to as ‘highCHmod’ genes) are associated with lower promoter accessibility (Figure 2I) and enhanced accumulation (Figure 2H) of H3K27me3 relative to those repressed genes with low levels of CH modification (referred to as ‘lowCHmod’ genes) (Figure 2F–J, Figure 2—figure supplement 1F-G). Gene ontology (GO) analysis indicates that both sets of genes that are repressed as PC differentiation proceeds are associated with development, and that the genes that do not accumulate 5mCH and 5hmCH encode preferentially proteins related to cell morphogenesis and axonal projection (Figure 2J). Although the data for highCHmod genes is consistent with transcriptional inhibition through both polycomb repressive complexes (Li et al., 2018) and MeCP2 binding (Gabel et al., 2015), our data do not identify the mechanisms responsible for repression of the lowCHmod gene class that fail to accumulate repressive CH marks over the gene body as they mature. To discover the mode of repression for these genes will require close examination of intergenic regulatory domains and associated transcriptional repressors and epigenetic marks.

### Developmentally activated PC expressed genes

We identified 432 genes that increase in expression as PCs differentiate (Figure 2A, Supplementary file 1). In general, these genes encode proteins known to be important for the mature functions of PCs, including calcium ion buffering and transport, regulation of the inositol triphosphate signaling pathway, and RNA splicing (Figure 2B). The chromatin landscape of most genes whose expression increases during PC differentiation is similar to those that are constitutively transcribed (e.g. *Atp2a3*, *Cep76*; Figure 1G). Their promoters are highly accessible (Figure 2D and K–L), they have high levels of H3K4me3 (Figure 2E), their gene bodies have low levels of 5mCG and elevated levels of 5hmCG (Figure 2C), and they do not accumulate 5mCH or 5hmCH (Figure 2N).

### Loss of 5mC and 5hmC in active genes during PC differentiation

**Figure 3.**
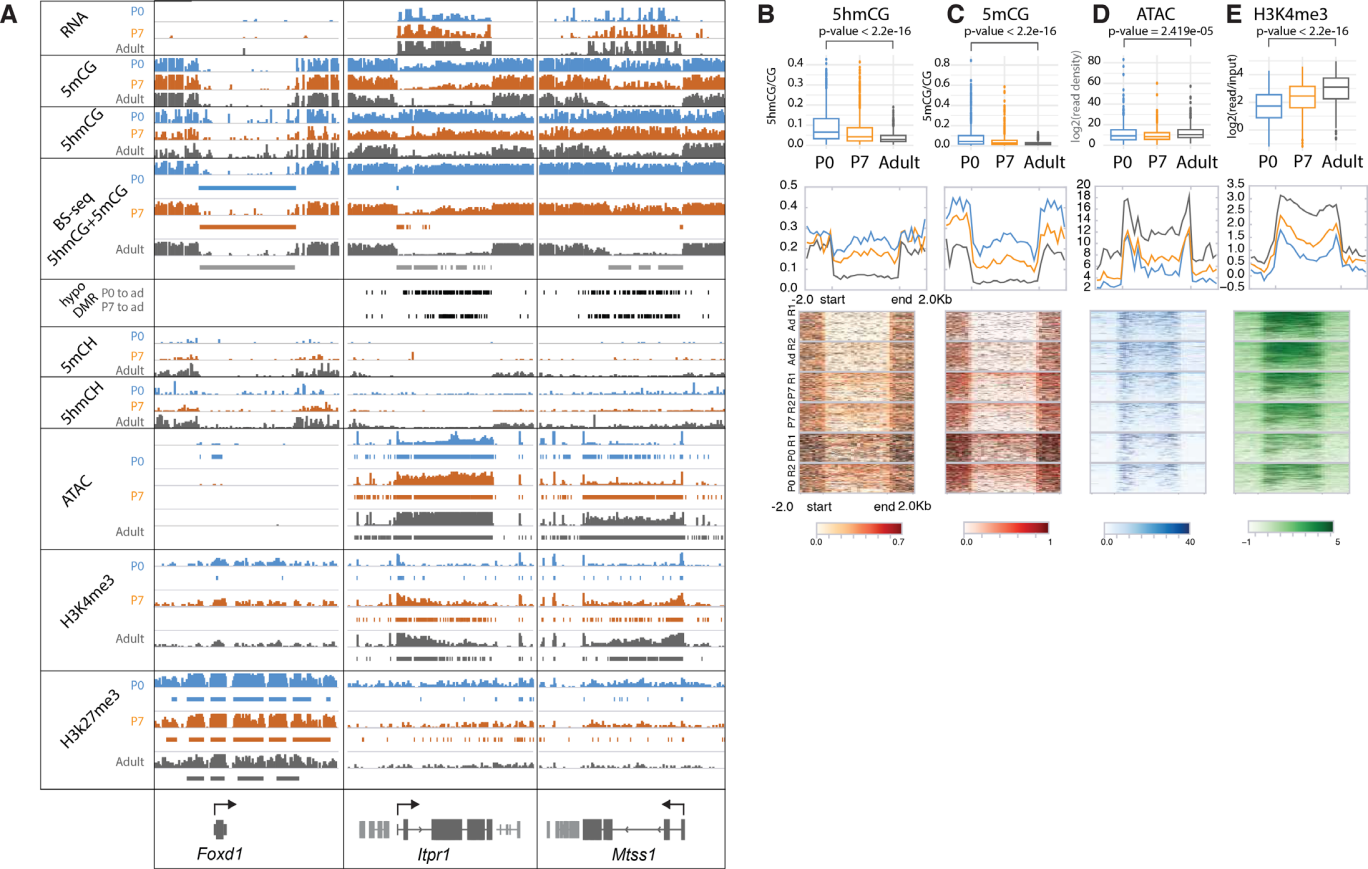
Loss of 5mCG and 5hmCG in a novel class of epigenetically regulated highly expressed Purkinje-specific genes. (**A**) Integrated genome viewer (IGV) representation of example regions of inactive DNA methylation valley (DMV) (Foxd1) and active Purkinje cell (PC)-specific DMVs (*Itpr1*, *Mtss1*). Bisulfite sequencing (BSSeq) tracks shows combined levels of mCG and hmCG ranging from 0 to 1. Bars under BSSeq tracks are computationally identified DMVs, black bars denote hypo-DMRs (differentially methylated regions) in adult, compared to P0 or P7. Bars under assay for transposase-accessible chromatin sequencing (ATACSeq), H3K4me3, and H3K27me3 tracks denote broad peaks of signal enrichment. (**B–E**) 5hmCG/CG (**B**), 5mCG/CG (**C**), ATACSeq log2(read density) RPKM enrichment (**D**) and H3K4me3 log2(input normalized) RPKM enrichment (**E**) quantification over DMVs at each timepoint. Boxplots show mean value per DMVs, the test for significance is Wilcoxon. Metagene plots show mean value ±2 kb around the DMV region, regardless of gene directionality. Heatmaps show data summarized in the metagene plots.

Our data reveal additional, surprising features that have not been documented in postmitotic cells. Thus, in a small subset of highly expressed genes (e.g. *Cep76*, *Itpr1*, *Mtss1*) there is a profound loss of both 5hmCG and 5mCG during the terminal, postmitotic stage of PC differentiation (Figures 1F and 3). To investigate the apparent demethylation over this class of genes, we computationally identified DNA methylation valleys (DMVs) as previously described (Jeong et al., 2014; Mo et al., 2015; Xie et al., 2013). In brief, DMVs were characterized by filtering undermethylated regions (UMRs) for length (>5 kb) and merging any regions within 1 kb (Burger et al., 2013; Figure 3—figure supplement 1A-B, Supplementary file 2). This revealed multiple regions longer than 15 kb, an unusual finding given that the average length of DMVs is ~5 kb (Figure 3—figure supplement 1D-E; Jeong et al., 2014). As expected from previous studies, a small number of these genes are inactive, fully demethylated, inaccessible, and covered by high levels of H3K27me3 repressive marks (Figure 3A, Foxd1, Figure 3—figure supplement 1C). These genes acquire their characteristics early in development, and their epigenetic features are stable.

**Figure 3—figure supplement 1.**
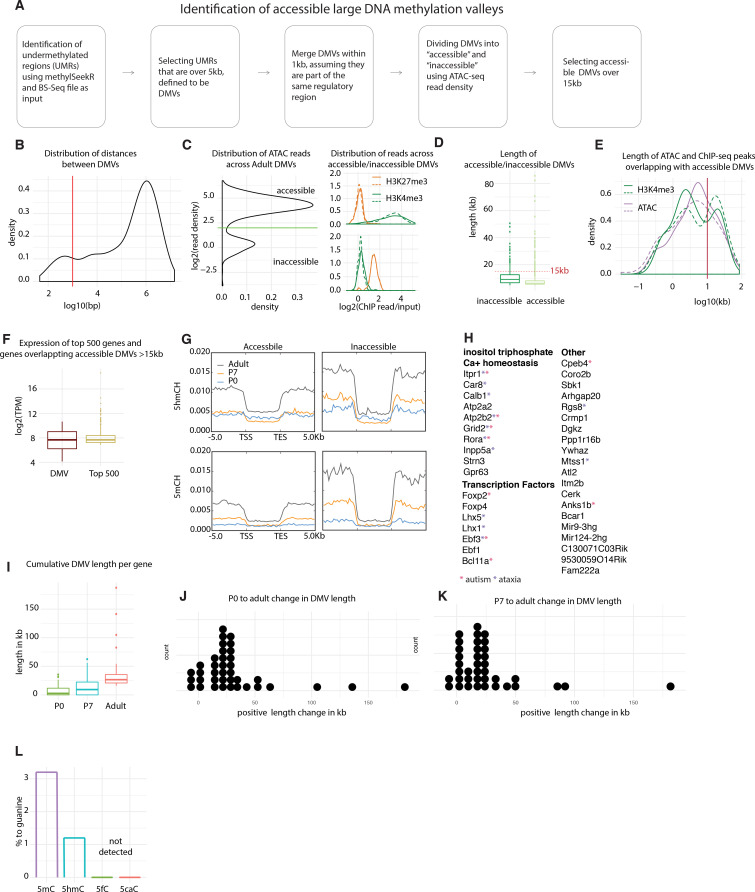
Characterization of Purkinje-specific DNA methylation valleys (DMVs). (**A**) Schematic of DMV identification and filtering. (**B**) Distribution of distances between DMVs. DMVs within 1 kb of each other are assumed to be part of the same regulatory region and therefore merged. (**C**) Assay for transposase-accessible chromatin sequencing (ATACSeq) read density over DMVs shows a bimodal distribution, segregating DMVs into accessible and inaccessible (left panel). Inaccessible DMVs show enrichment for H3K27me3 reads, while accessible DMVs show enrichment for H3K4me3 reads (right panel, dotted and straight lines designate replicates). (**D**) Distribution of lengths of accessible and inaccessible DMVs. (**E**) Length of broad peaks of ATACSeq and H3K4me3 chromatin immunoprecipitation sequencing (ChIPSeq) overlapping large accessible DMVs. (**F**) Expression of the top 500 most expressed genes in Purkinje cells (PCs) and the genes associated with DMVs. (**G**) DMVs do not accumulate any modifications in CH context. (**H**). Genes associated with DMVs, red star denotes role in autism, gray in ataxia (**I**). Cumulative length of DMVs per gene. (**J–K**) Change of DMV length between P0 and adult (**J**) and P7 and adult (**K**). (**L**) Mass spectrometry analysis of genomic DNA from adult PCs.

The majority of very large DMVs in PCs, however, arise during differentiation through loss of 5mC and 5hmC over active, highly expressed genes as PCs mature (Figure 3, *Itpr1*, *Mtss1*). In these genes, the timing of changes in DNA methylation, chromatin accessibility, and dynamic histone marks varies from gene to gene, but the overall progression of events associated with their expression is similar (Figure 3). At birth, these genes are actively transcribed (Figure 3A), 5hmCG is enriched in the gene body (Figure 3B) and 5mCG is depleted (Figure 3C), their promoters are accessible (Figure 3D), and H3K4me3 is present over their promoters (Figure 3E). These epigenetic properties resemble moderately expressed genes in many cell types. As differentiation proceeds and transcription increases, there is a gradual decrease in both 5mCG, 5hmCG, and H3K27me3 histone marks, and ATAC accessibility and H4K4me3 marks spread from the promoter into the gene body. To further support these data, we have included analysis of independent BSSeq experiments that detect the combined levels of 5mC and 5hmC (Figure 3A, BSSeq). These data also reveal that hypo-DMRs (differentially methylated regions) present at P0 expand and fuse as PCs lose both methylation and hydroxymethylation to form the very large DMRs that are characteristics of these genes (Figure 3A, hypo-DMR track). As shown in Figure 3B–E and Figure 3—figure supplement 1I-K, quantitation of these features demonstrates that that loss of DNA methylation and hydroxymethylation occurs over the entire gene body for these genes as H3K4me3 activating histone marks accumulate and ATAC accessibility increases.

Interestingly, many of the active genes associated with broad DMVs are PC-specific and highly expressed (Figure 3—figure supplement 1F). GO analysis indicates that they are involved in the inositol triphosphate/calcium signaling pathways, and a subset is associated with ataxia and autism (Figure 3—figure supplement 1H). They do not accumulate modified cytosines in CpH context (Figure 3—figure supplement 1G). These data provide strong evidence that DNA demethylation can occur in postmitotic neurons, and that it is enhanced in a specific class of genes that are very highly expressed and functionally important. Although our mass spectroscopic analysis of genomic DNA from differentiating PCs (Figure 3—figure supplement 1L) failed to detect 5fC or 5caC, their involvement as transient intermediates in the loss of 5mC and 5hmC cannot be ruled out because the small fraction of the genome covered by this gene class may preclude their detection (Guo et al., 2011; He et al., 2011; Ito et al., 2011).

### Loss of 5mC and 5hmC in putative regulatory sites during PC differentiation

Previous studies of enhancers and other regulatory sites have established that their activation is accompanied by increased ATAC accessibility and loss of DNA methylation (Lio et al., 2016). To determine whether loss of DNA methylation can occur in putative regulatory sites in addition to large DMRs in postmitotic PCs, we used an established computational method (Burger et al., 2013) to identify small regions with statistically significant ATACSeq signal enrichment (Figure 2K–N). We then employed differential accessibility analysis to divide those regions into two groups based the magnitude (log2 fold change >4) and significance (p < 0.01) of their changes during PC differentiation. Those that became more accessible between P0 and adult (log2 fold change >4) experienced a ‘gain’ and those whose accessibility diminished during this time are characterized by a ‘loss’. As anticipated, those ATAC peaks that gain accessibility lose both 5hmC and 5mC as cells progress from P0 to adult (Figure 2N, Gain section) whereas no significant changes in methylation at ATAC peak centers occur in sites that lose accessibility (Figure 2N, Loss section). Although these ATAC peaks have not been identified as active enhancers, it is noteworthy that the transcription factor motifs found in these different classes of ATAC sites are distinct (Figure 2—figure supplement 1H). These findings are consistent with prior studies indicating that enhancer activation is accompanied by enhanced ATAC accessibility and DNA demethylation (Lio et al., 2016). In this case, however, DNA demethylation does not require cell division.

### PC-specific *Tet1*, *Tet2*, *Tet3* triple knockout mouse lines

**Figure 4.**
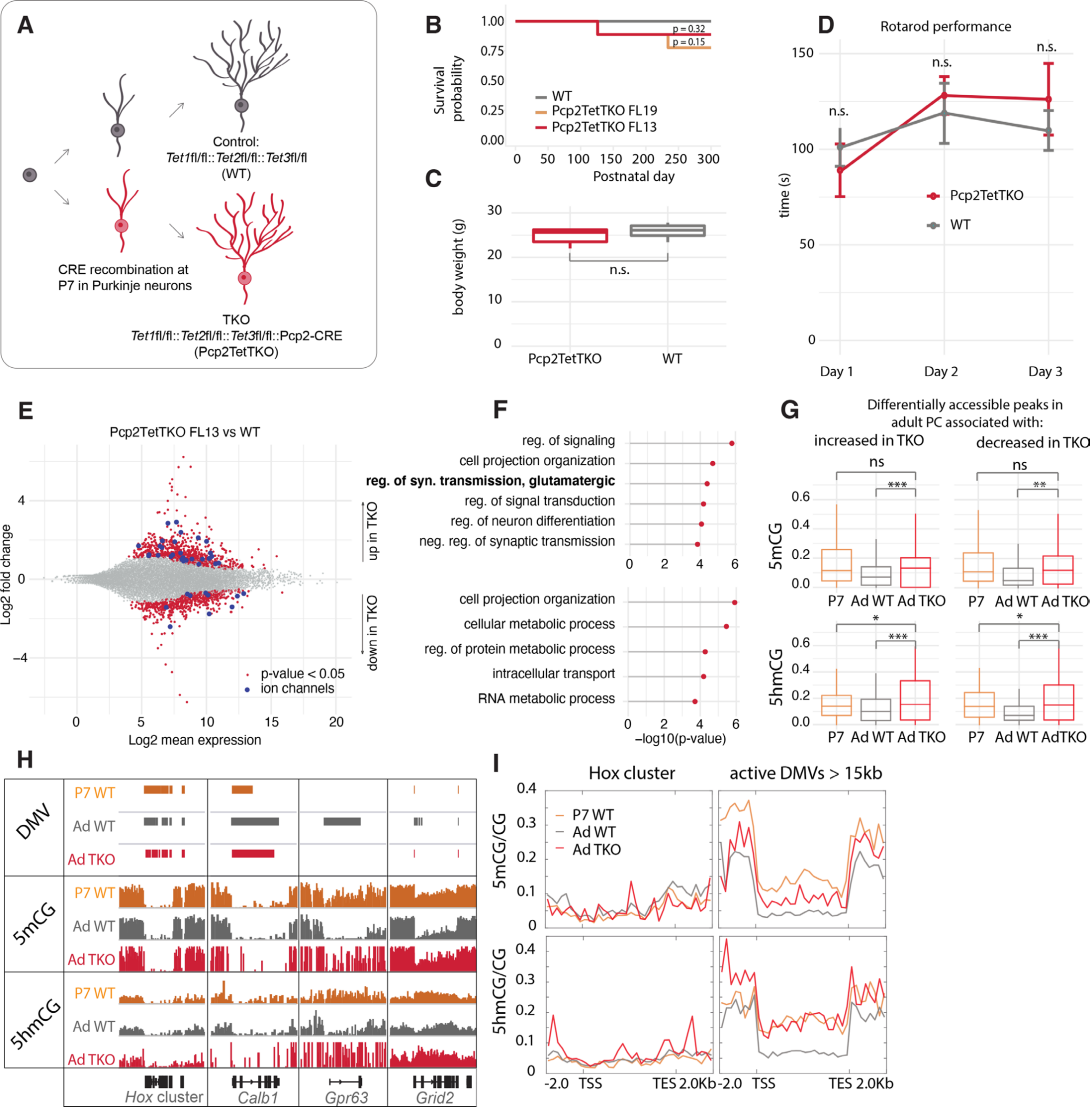
Loss of Tet activity leads to impaired gene expression regulation in Purkinje cells (PCs). (**A**) Schematic of experimental design to probe the triple Tet1, Tet2, Tet3 knockout effect in adult PCs. (**B**) Kaplan-Meier curve representing the survival fraction of wild-type (WT) (n = 8) and PC-specific triple knockout (Pcp2TetTKO) (Founder13 n = 8, Founder19 n = 8) mice since date of birth. (**C**) Body weight of WT and Pcp2TetTKO at 8 weeks. (**D**) Rotarod evaluation of motor skills in WT and Pcp2TetTKO at 8 weeks. (**E**) MA plot representing differential gene expression analysis between WT and Pcp2TetTKO. Navy dots represent genes with p-value < 0.05, red dots – ion channels. (**F**) Gene ontology analysis of statistically significant differentially expressed genes. Top panel shows categories of genes with increased expression in Pcp2TetTKO, bottom panel shows genes with decreased expression in Pcp2TetTKO. (**G**) Levels of 5hmCG/CG and 5mCG/CG over differentially accessible peaks only present in adult PCs (compared to P0). (**H**) Integrated genome viewer (IGV) representation of example DNA methylation valleys (DMVs) affected by Pcp2TetTKO. Hox cluster and Calb1 show minor changes in length as they are established before the P7 onset of Cre. Gpr63 and Grid2 show significant reduction in length as they are established after P7. Solid bars represent DMVs identified at each condition. (**I**) Quantification of 5hmCG/CG and 5mCG/CG over the two classes of DMVs – Hox cluster and large active DMVs.

Despite convincing evidence that Tet-mediated active DNA demethylation can occur in mouse ESCs through the TDG-BER pathway (He et al., 2011; Weber et al., 2016), evidence that this can occur in vivo has been difficult to obtain because such a proof requires loss or complete inhibition of all three Tet oxidases in a single cell type after cells have exited the cell cycle permanently. To provide this evidence, we generated PC-specific *Tet1/Tet2/Tet3* triple knockout (Pcp2TetTKO) mouse lines (Figure 4A). We chose to drive Cre recombinase expression using an engineered *Pcp2* BAC employed previously to generate accurate Cre driver lines (Gong et al., 2007) because the onset of expression of the *Pcp2* gene and the corresponding BAC vector occurs approximately 1 week after birth as the cerebellum enters its terminal phase of development and maturation. The Pcp2Cre BAC was introduced by pronuclear injection into ova from females carrying floxed alleles of all three Tet genes (Figure 4—figure supplement 1A-B) yielding several founder lines. PCR analysis of the floxed regions of each Tet gene in purified PC genomic DNA, and RNASeq analysis confirmed the deletion of exons from all three TET proteins (Figure 4—figure supplement 1C-D). The lines chosen for analysis displayed no gross motor phenotype as assessed by rotarod performance, survival was normal, and recombination activity was specific to PCs (Figure 4B–D, Figure 4—figure supplement 2A-D). The deletion of the Tet proteins did not affect the expression of *Itpr1* significantly, and we confirmed that it could still be used for purification of PCs (Figure 4—figure supplement 1E-F). Evaluation of the quality control metrics of the datasets showed strong correlation coefficients and enrichment for Purkinje markers (Figure 4—figure supplement 1G-H). Cre recombinase activity, and thus recombination in the Tet genes, began at approximately P7 more than 2 weeks after PCs have completed their last division (Figure 4—figure supplement 2A-D). These lines, therefore, allow assessment of the consequences of loss of Tet activity during the rapid phase of somatic and dendritic growth in postmitotic, differentiating Purkinje neurons.

**Figure 4—figure supplement 1.**
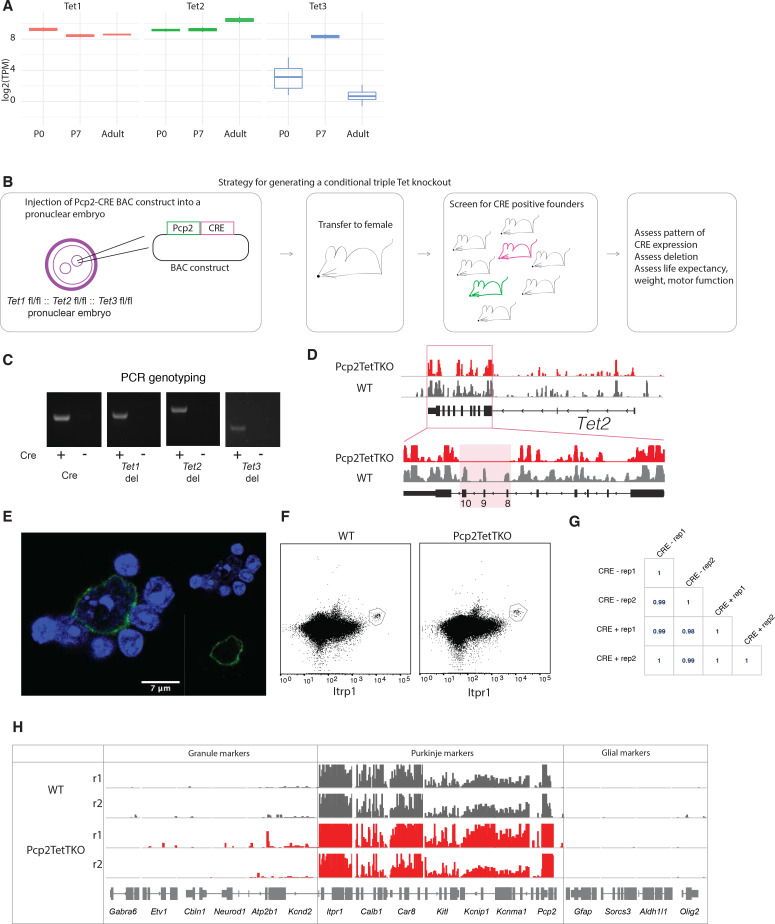
Generation of Pcp2 cre/wt:: *Tet1* del/del:: *Tet2* del/del:: *Tet3* del/del (Purkinje cell-specific triple knockout [Pcp2TetTKO]) mouse founder lines. (**A**) Expression levels of Tet1, Tet2, and Tet3 proteins at P0, P7, and adult developmental stages of Purkinje cells. (**B**) Schematic of Pcp2TetTKO generation via pronuclear injection. (**C**) PCR genotyping for CRE, *Tet1* deletion, *Tet2* deletion, and *Tet3* deletion in adult Pcp2TetTKO Purkinje genomic DNA. (**D**) Deletion of *Tet2* exons evident in nuclear RNASeq Pcp2TetTKO. (**E**) Example of Pcp2TetTKO nuclei stained with Itpr1 post-dissociation and pre-sorting, counterstained with DAPI, a heterochromatin marker. (**F**) Example fluorescence-activated nuclear sorting (FANS) plot of adult Pcp2TetTKO nuclei. (**G**) Pearson correlation between TKO and control. (**H**) Integrated genome viewer (IGV) representation of Purkinje-specific markers (*Itpr1, Calb1, Car8, Kitl, Kcnip1, Kcnma1, Pcp2*) enrichment and depletion of granule (*Gabra6, Etv1, Cbln1, Neurod1, Atp2b1, Kcnd2*) and glial (*Gfap, Sorcs3, Aldh1l1, Olig2*) markers in Pcp2TetTKO and wild type (WT).

**Figure 4—figure supplement 2.**
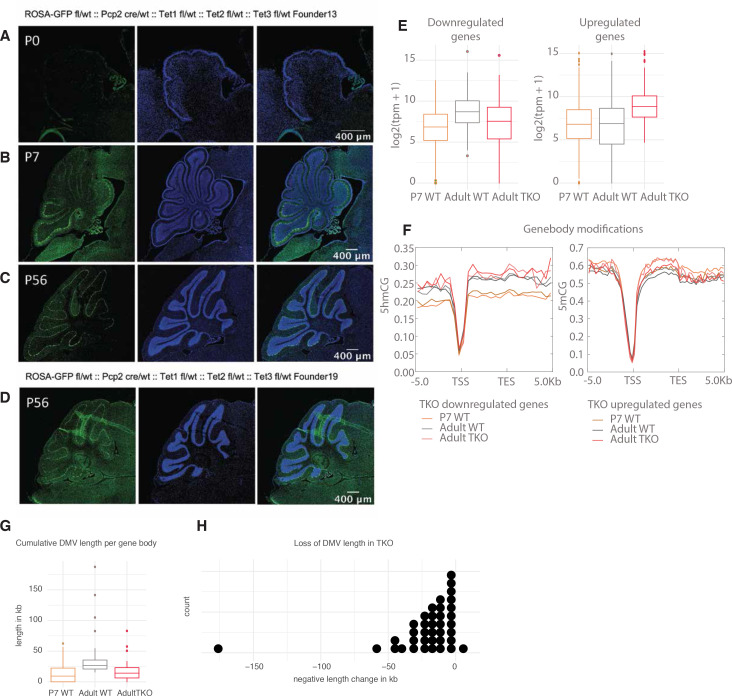
Cytosine dynamics in Purkinje cell-specific triple knockout (Pcp2TetTKO). (**A-C**) Pcp2 cre/wt:: Tet1 del/del:: Tet2 del/del:: Tet3 del/del mice were crossed to ROSA26-eGFP reporter line to evaluate CRE expression during development. Panels represent murine cerebella at P0 (**A**), P7 (**B**), and P56 (**C**) timepoints in FL13, stained with anti-GFP antibody. (**D**) Anti-GFP staining in P56 FL19. (**E**) Expression of genes with increased and decreased expression in Pcp2TetTKO (boxplots in left panel, heatmaps in right panel). (**F**) Metagene plot of mean mCG and hmCG modifications over promoters and gene bodies of genes with increased or decreased expression in Pcp2TetTKO. (**G**) Cumulative DNA methylation valley (DMV) length per gene body between Pcp2TetTKO and wild type (WT). (**H**) Change in cumulative DMV length between adult Pcp2TetTKO and WT.

### Altered transcription of PC expressed genes in Pcp2TetTKO

Loss of function studies of *Tet1*, *Tet2*, *Tet3* and combinations thereof in ESCs and lymphocyte lineages have established that 5hmC plays a direct role in transcriptional regulation as a consequence of Tet-mediated replication-dependent loss of 5mC in enhancers, promoters, and gene bodies. To determine whether disruption of ongoing 5hmC accumulation in postmitotic PCs results in altered transcriptional regulation despite the inability to utilize passive DNA demethylation as a regulatory mechanism, we compared the transcriptome of adult Pcp2TetTKO mice with floxed Cre-negative littermates (WT). Using p < 0.05, log2 fold change of >0.5 in either direction to filter these data, we identified 721 genes whose transcription increases in the Pcp2TetTKO PCs and 548 genes with decreased levels of expression (Figure 4E, Supplementary file 3, Figure 4—figure supplement 2E-F). The large number of genes whose expression is impacted in the Pcp2TetTKO PCs is reminiscent of magnitude of changes documented in neurons in response to loss of MeCP2 (Chahrour et al., 2008) and other proteins involved nuclear organization and chromatin structure (Hyun et al., 2017). GO analysis of these two classes of genes revealed differences in the biological categories represented (Figure 4F). It is noteworthy that genes whose expression increases in the Pcp2TetTKO PCs are enriched for functions involved in terminal developmental events such as cell projection organization, synaptic transmission, and neuron differentiation. These include many ion channels, and receptors that should impact the fine-tuned physiology of PCs (Figure 4E–F, dark blue dots, Supplementary file 3), although given the complex firing patterns and action potential (AP) waveforms characteristic of PCs, it is difficult to predict the functional consequences of these changes. In contrast, genes whose expression is less in Pcp2TetTKO PCs are primarily involved in homeostasis and metabolic control. These are enriched in DNA transmembrane receptors and nucleic acid-binding proteins that may help regulate PC metabolism in response to external signals.

### DNA demethylation in postmitotic PCs requires 5hmC

To assess the loss of 5mC and 5hmC in differentiating PCs reflects a direct role of TET proteins in transcriptional regulation as a consequence of loss of 5mC in enhancers, promoters, and gene bodies, we first identified candidate regulatory domains by comparative analysis of P0 and adult ATACSeq data to detect changes in chromatin accessibility that occur during PC differentiation. We then focused on those regions associated with genes that are transcriptionally impacted in the Pcp2TetTKO. As shown in Figure 4G, PC regulatory sites that become more accessible as differentiation proceeds lose both 5mCG and 5hmCG between P7 and adult whether their expression increases or decreases in the Pcp2TetTKO cells. The increased 5mCG and 5hmCG evident over these sites in the Pcp2TetTKO relative to WT PCs indicates clearly that loss of both 5mCG and 5hmCG at these sites requires continued Tet oxidase activity.

The requirement for continued 5hmC production in DNA demethylation is particularly evident from analysis of those very highly expressed genes that lose both 5mCG and 5hmCG over the gene body as differentiation proceeds (Figure 3). Genes with very large DMVs that are acquired early in development, for example, Hox cluster genes and Calb1, are not impacted in the Pcp2TetTKO PCs (Figure 4H, I). This is expected because DNA demethylation for these genes is evident before recombination and loss of Tet oxidase function occurs in the Pcp2TetTKO lines at approximately postnatal day 7 (Figure 4—figure supplement 2A-D). In contrast, for those genes that acquire large DMVs between P7 and adult, for example *Gpr63* and *Grid2*, the loss of DNA methylation that is required for formation of the DMVs is strongly decreased (Figure 4H, I). As shown in the metagene plots of DNA displaying 5mCG and 5hmCG levels of this class of genes over the gene body (Figure 4I, right panel), the loss of both 5mCG and 5hmCG that occurs between P7 and adult is strongly impacted. Thus, the low levels of 5hmCG and 5mCG characteristic of this class of genes is not attained in the absence of Tet oxidase activity.

Formation of large DMVs in PC genes differs in detail depending on the timing and rate of transcription and the size of the gene. *Gpr63* is strongly activated during postnatal life to become one of the most actively transcribed genes in adult PCs and it becomes nearly completely demethylated over the entire 46 kb of its gene body. *Grid2* is a very long gene (~1.44 Mb) whose transcription also increases as differentiation proceeds. The *Grid2* DMV also fails to develop fully in the Pcp2TetTKO (Figure 4H). In this case, the large DMV develops over only the promoter and initial 5’ region of the gene. In these two cases, and in other genes of this class, the growth of the DMV is preceded by the accumulation of 5hmC, DNA demethylation is initiated at the 5’ end of the gene, and it spreads toward the 3’ end as differentiation proceeds (Figure 4H). To understand better this variation in DNA demethylation in individual genes, we calculated the difference in the length of large DMVs over each gene in this class in the WT and Pcp2TetTKO PCs, and plotted these values as the negative length change (Figure 4—figure supplement 2G, H). These data revealed a large variation in the length of the DMV that does not form in the Pcp2TetTKO PCs. We believe this reflects a complex relationship between the timing at which transcription is initiated, the rate of transcription over the entire gene body, the local activity of Tet oxidase within the gene as differentiation proceeds, and the timing of Tet oxidase loss from each cell following recombination at the *Tet1*, *Tet2*, and *Tet3* loci.

Taken together, these data demonstrate that loss of DNA methylation occurs over specific subsets of regulatory sites and transcription units in postmitotic Purkinje neurons. This loss of 5mCG requires continued production of 5hmC by Tet1, Tet2, and Tet3. The simplest interpretation of these data is that Tet-mediated active demethylation can occur in neurons as a result of continued oxidation of 5hmC to 5fC and 5caC, followed by their removal through the BER pathway.

## Discussion

Since the discoveries that 5hmC is present at high levels in mammalian neuronal genomes (Kriaucionis and Heintz, 2009) and that it is produced from 5mC by the Tet oxidases (Tahiliani et al., 2009), its possible roles as a stable epigenetic mark and as an intermediate in DNA demethylation have been intensively investigated (Wu and Zhang, 2017). The present study adds to a growing body of work demonstrating that 5hmC plays a critical role in development and function of the nervous system. A central finding of this study is that continued Tet activity and 5hmC accumulation during PC differentiation is necessary for acquisition of their refined transcriptional and epigenetic properties. It is noteworthy that these phenotypes are evident despite loss of Tet function in the Pcp2TetTKO late in PC differentiation. It seems likely based on these findings and the gradual accumulation of 5hmC evident in our data that there is a continual requirement for 5hmC as a driver for epigenetic remodeling in most differentiating, postmitotic neurons. Although studies of neural stem cells (Li et al., 2015; Xu et al., 2012), cerebellar granule cells (Zhu et al., 2016), and embryonic brain development have suggested that 5hmC plays a significant role in neural progenitors and developing granule cells, a continual requirement for 5hmC may be particularly important for PCs, pyramidal cells, and other long range projection neurons whose differentiation program includes postmitotic development of sophisticated morphological features and abundant synapses.

A second principle that has emerged from our studies is that Tet-mediated active DNA demethylation occurs in postmitotic neurons. Our finding that loss of both 5mC and 5hmC in a specific subset of highly expressed gene bodies and ATAC accessible regulatory sites requires Tet oxidase activity provides critical in vivo evidence supporting studies in vitro (Weber et al., 2016) and in cultured cells (He et al., 2011) that have defined this pathway. For some genes (*Gpr63*, *Grid2*) the progression toward loss of modified C is easily appreciated. In these cases, conversion of 5mCG to 5hmCG over the gene body is clearly evident between P0 and P7, and continued Tet activity is required to remove both residual 5mCG and 5hmCG to result in DNA demethylation in the adult. Although we have been unable to detect 5fC and 5caC as transient intermediates in this process (Figure 3—figure supplement 1L), this is not surprising given the very small fraction of the PC genome that undergoes active DNA demethylation and the small amounts of genomic DNA that can be obtained from this very rare cell population. Identification of additional components of the of the DNA demethylation pathway that is occurring in PCs will require further genetic studies disrupting candidate genes in the TDG-BER pathway. Since important non-cell autonomous events are required for PC differentiation (Leto et al., 2016), we believe that additional PC-specific knockouts that are restricted to postmitotic cells will be most informative.

An important question arising from these findings is which cells in the nervous system require Tet-dependent active DNA demethylation to complete their developmental programs. Given these data, it seems likely other large neurons with complex and prolonged postmitotic differentiation programs share this requirement. Furthermore, reports that remodeling of DNA methylation may occur in response to a wide variety of stimuli in adult mice (Kaas et al., 2013; Rudenko et al., 2013) suggest that additional studies of Pcp2TetTKO and other precisely constructed mouse models will help elucidate possible functions of 5hmC in neuronal plasticity.

A third advance reported here is the collection of very high-resolution data to understand details of the relationships between DNA methylation and hydroxymethylation, chromatin accessibility, and chromatin organization in a single complex neuronal cell type as it develops from a primitive neuronal precursor to a fully articulated adult neuron. These data have highlighted several features of epigenetic development that remain to be explored including the definition of two distinct relationships between non-CG DNA methylation/hydroxymethylation and transcriptional repression, and the mechanism for selection of specific genes as substrates for active DNA demethylation. Taken together, our data demonstrate that disruption of continued 5hmC formation in the Pcp2TetTKO postmitotic PCs leads to altered transcription of late expressed genes, including a set of ion channels that may be responsible for refining their adult electrophysiological properties.

Although our data reinforce prior studies of the general relationships between DNA methylation, transcription, and chromatin structure, we note that there are many exceptions that require further investigation. One intriguing example is the very different relationships between genes and de novo methylation in CG and non-CG contexts (Lister et al., 2013). In PCs, as in other neurons, substantial enrichment of 5hmCG relative to its general accumulation genome wide occurs in active transcription units, suggesting a mechanism that targets Tet activity to these regions (Figure 2C). While accumulation of 5mCH and 5hmCH can occur within a repressed transcription unit with very nice discrimination between the gene and its surroundings (e.g. Pitpnc1), there are many regions of 5mCH and 5hmCH accumulation that either partially overlap with a gene, or that are present in intergenic regions (e.g. *Myo5b*/*Scarna17*/*Lipg* locus). In all of these regions, however, 5hmC accumulation directly reflects 5mCH levels suggesting 5hmCH localization reflects the pattern of de novo DNA methylation rather than targeted Tet activity. We hope that continued analysis of these datasets will provide a stimulus for additional studies of the mechanisms determining the relative distributions of these important events.

### Concluding remarks

The data we have reported here advances our understanding of three major functions for 5hmC in the nervous system. Based on extensive studies of the requirements for Tet-mediated replication-dependent passive DNA demethylation in mESCs, the maturation of the germline, and developing lymphocyte lineages, it is probable that 5hmC is required to provide accessibility to important regulatory sites as neuronal progenitors exit the cell cycle and begin differentiation. The lack of DNA methylation we have observed at P0 over transcription factor genes (*Lhx5*, *Lhx1*, *Ldb*) that are expressed immediately after PCs exit from the cell cycle suggests that this may result from passive demethylation in dividing progenitors. Further support comes from the observation that DNA demethylation has been observed in comparisons of 5mC and 5hmC levels in the frontal cortex of fetal versus adult mouse and human brains, and the finding that a fraction of these loci retain their methylation status in *Tet2*^-/-^ mice (Lister et al., 2013). A second function discovered in mature neurons involves stable accumulation of 5hmCG within active genes which helps to reverse the repressive effects of MeCP2 in a process we refer to as functional demethylation (Mellén et al., 2017). The finding that elevated 5hmC remains within genes that are repressed during PC differentiation (Figure 2C) adds to this model the fact that functional demethylation is not sufficient to maintain gene expression. Finally, we report here that Tet-mediated active DNA demethylation is required for proper expression of a subset of highly expressed PC-specific genes, adding a third important function for 5hmC in the nervous system. These findings place additional emphasis on investigation of ongoing functions of the TDG-BER pathway as elements of normal neuronal development rather than responses to accumulating DNA damage. Given these data and recent studies linking Tet mutations to neurodegenerative disease (Cochran et al., 2020; Marshall et al., 2020), it is evident that further exploration of each of these functions in the context of development, aging, and degeneration will continue enhance our understanding of 5hmC and the brain.

## Materials and methods

**Key resources table:** 

Reagent type (species) or resource	Designation	Source or reference	Identifiers	Additional information
Strain, strain background (*Mus musculus*)	Wild type	Jackson Labs	RRID:IMSR_JAX:000664	
Strain, strain background (*Mus musculus*)	*Tet1* fl/fl:: *Tet2* fl/fl:: *Tet3* fl/fl	Anjana Rao		
Strain, strain background (*Mus musculus*)	*Tet1* fl/fl:: *Tet2* fl/fl:: *Tet3* fl/fl:: Pcp2-CRE	This paper		This line was generated as described in the Materials and methods section ‘Pcp2TetTKO murine strain generation’
Recombinant DNA reagent	Pcp2-CRE (BAC construct)	GENSAT	RP24-186D18	
Antibody	Anti-Itpr1 (mouse monoclonal)	Origene	Origene: TA326547	(1:500)
Antibody	Anti-Itpr1 (mouse monoclonal)	Abcam	Abcam: ab190239	(1:500)
Antibody	Anti-Calb1 (rabbit polyclonal)	Immunostar	RRID: AB_572222Immunostar: 24,427	(1:500)
Antibody	Anti-H3K27me3 (rabbit polyclonal)	Active motif	RRID: AB_2561020Active motif: 39,155	(4 µL)
Antibody	Anti-H3K4me3 (rabbit polyclonal)	Active motif	RRID: AB_2615077Active motif: 39,159	(4 µL)
Antibody	Anto-GFP (chicken polyclonal)	Abcam	Abcam: ab13970	(1:500)
Sequence-based reagent	Raw and processed sequencing data	This paper	GSE166423	The sequencing data was generated as described in the Materials and methods section ‘Sequencing methods’
Commercial assay or kit	AllPrep FFPE	Qiagen	Qiagen: 80,234	
Commercial assay or kit	Low Cell ChIPSeq kit	Active motif	Active motif: 53,084	
Commercial assay or kit	Ultralow MethylSeq with Trumethyl OxBS	Tecan Genomics	Tecan Genomics: 0414–32	
Commercial assay or kit	Nextera library prep kit	Illumina	Illumina: FC-131–1024	
Commercial assay or kit	Bioanalyzer Pico	Agilent	Agilent: 5067–1513	
Commercial assay or kit	TapeStation High sensitivity D1000	Agilent	Agilent: 5067–5593	

### Animals

Wild-type C57BL6/J (RRID:IMSR_JAX:000664) were obtained from Jackson Laboratories. Animals were maintained on a 12 hr light/12 hr dark cycle with food and water ad libitum. Animal protocols were approved by the Rockefeller University Institutional Animal Care and Use Committee, in accordance with the US National Institutes of Health Guide for the Care and Use of Laboratory Animals.

### Pcp2TetTKO murine strain generation

Tet1fl/fl::Tet2fl/fl::Tet3fl/fl animals were a gift from Anjana Rao. We used a previously characterized Pcp2-CRE BAC (RP24-186D18) construct from the GENSAT project (Gong et al., 2007; Gong et al., 2003) to create a conditional TKO in PCs. Pronuclear injections of the BAC construct into zygotes were performed at the Transgenic and Reproductive Technology Center at the Rockefeller University.

### Cell type-specific nuclei isolation

#### Nuclei isolation

Nuclei isolation protocol was followed as described (Xu et al., 2018). Briefly, mouse cerebella were dissected and flash-frozen using liquid nitrogen. To isolate nuclei, tissue was thawed on ice for 30 min and then transferred to 5 mL of homogenization buffer (0.25 M sucrose, 150 mM KCl, 5 mM MgCl_2_, 20 mM Tricine pH 7.8, 0.15 mM spermine, 0.5 mM spermidine, EDTA-free protease inhibitor cocktail, 1 mM DTT, 20 U/mL Superase-In RNase inhibitor, 40 U/mL RNasin ribonuclease inhibitor). Tissue was homogenized by 30 strokes of loose (A) followed by 30 strokes of tight (B) glass dounce. Homogenate was supplemented with 5 mL of a 50% iodixanol solution (50% Iodixanol/Optiprep, 150 mM KCl, 5 mM MgCl2, 20 mM Tricine pH 7.8, 0.15 mM spermine, 0.5 mM spermidine, EDTA-free protease inhibitor cocktail, 1 mM DTT, 20 U/mL Superase-In RNase inhibitor, 40 U/mL RNasin ribonuclease inhibitor), and laid on a 27% iodixanol cushion. Nuclei were pelleted by centrifugation 30 min, 10,000 rpm, 4°C in swinging bucket rotor (SW41) in a Beckman Coulter XL-70 ultracentrifuge. The nuclear pellet was resuspended in homogenization buffer.

#### Nuclei labeling and sorting

Nuclei were fixed with 1% formaldehyde for 8 min at RT with mild agitation. The crosslinking reaction was quenched with 0.125 M glycine for 5 min at RT. Nuclei were pelleted at 1000 g, 4 min, 4°C, and then washed two times with Wash Buffer (PBS, 0.05% Triton X-100, 50 ng/mL BSA, 1 mM DTT, 10 U/µL Superase-In RNase Inhibitor). Nuclei were blocked with Block Buffer (Wash Buffer with an additional 50 ng/mL BSA) for 30 min at RT, incubated with primary antibody for 1 hr at RT, and then washed three times with Wash Buffer with spins in-between washes as described above. Nuclei were then incubated in secondary antibody for 30 min at RT and washed three times with Wash Buffer. Primary and secondary antibodies were diluted in Block Buffer. Secondary antibodies were purchased from Life Technologies or Jackson Immunoresearch and were used at 1:500 dilution. Secondary antibodies from goat used: mouse Alexa488, rabbit Alexa594.

#### FACS

Nuclei were stained with DyeCycle Ruby to 20 µM final concentration. Nuclei were sorted using a BD FACSAria cell sorter using the 488 and 561 nm lasers. First, samples were gated using DyeCycle Ruby to select only singlets, then appropriate populations were gated based on their separation. Analysis was performed using FlowJo software. Qiagen Buffer PKD was added to the sorted nuclei and the samples were stored at –80°C.

### Immunohistochemistry

Mice were decapitated at P0 and P7, and the brains were dissected, and immersion fixed in 4% formaldehyde (w/v) overnight at 4°C. Adult mice were deeply anesthetized and then the brains were fixed by transcardiac perfusion with PBS followed by 4% formaldehyde. Brains were further fixed by immersion fixation in 4% formaldehyde overnight at 4°C. All brains were cryoprotected in 30% sucrose in PBS, embedded in OCT, and cut with a Leica CM3050 S cryostat into 20 µm sections. The sections were immediately mounted on slides and stored at –20°C. Antigen retrieval using sodium citrate buffer (10 mM sodium citrate, 0.05% Tween 20, pH 6.0) was performed by heating the slides to 95–100°C and then 10 min incubation in the microwave at the lowest power. The slides were cooled off to RT for 1 hr. The slides were washed in PBS and blocked with 3% BSA in PBS with 0.1% Triton X-100 for 30 min at RT. Primary antibody incubation was performed overnight at RT, washed with PBS, incubated with secondary antibody for 1 hr at RT, washed with PBS, stained with DAPI (1:10000) for 10 min at RT and washed three times with PBS. Slides were cover-slipped with Prolong Diamond mounting media. Images were acquired using a Zeiss LSM700 confocal microscope using the same acquisition settings for all samples. Further image analysis was done using FIJI.

### Sequencing methods

#### RNASeq

RNA and gDNA from fixed nuclei were purified using the Qiagen AllPrep FFPE kit with the following modifications. After DNA/RNA separation spin, the RNA-containing supernatant was removed and incubated at 65°C for 30 min, 70°C for 30 min, and 80°C for 15 min and then proceeded with the manufacturer’s protocol. gDNA was purified following the rest of the manufacturer’s protocol. RNA quality was determined using Agilent 2100 Bioanalyzer. Purified RNA was converted to cDNA and amplified using the Nugen Ovation RNA-Seq System V2. cDNA was fragmented to an average size of 200 bp using a Covaris C2 sonicator (intensity 5, duty cycle 10%, cycles per burst 200, treatment time 120 s). Libraries were prepared using the NEBNext Ultra DNA Library Prep Kit for Illumina with NEBNext Multiplex Oligos for Illumina. The quality of the libraries was assessed using the Agilent 2200 TapeStation system with D1000 High Sensitivity ScreenTape. Libraries were sequenced at The Rockefeller University Genomics Resource Center on the Illumina NextSeq 500 to obtain 75 bp paired-end reads.

#### ATAC-Seq

ATACSeq libraries were prepared as described (Buenrostro et al., 2013; Chen et al., 2016) with minor modifications; ~25 k fixed nuclei were incubated for 10 min in lysis buffer (10 mM Tris pH 7.5, 10 mM NaCl, 3 mM MgCl2, 0.1% NP-40). Nuclei were then resuspended in 50 µL 1× TD buffer containing 2.5 µL Tn5 enzyme from Nextera (Illumina) and incubated for 30 min at 37°C; 200 µL of reverse-crosslinking buffer (50 mM Tris-Cl, 1 mM EDTA, 1 % SDS, 0.2 M NaCl, 5 ng/mL proteinase K) was added to the samples and they were incubated overnight at 65°C with shaking. The samples were then purified using the QiaQuick MinElute columns (Qiagen). Libraries were amplified by PCR using the Q5 High Fidelity Polymerase (NEB) for 12 cycles with barcoded primers. Libraries were size-selected with AMPure XP beads (Beckman Coulter). Libraries were sequenced at The Rockefeller University Genomics Resource Center on Illumina NextSeq 500 to yield 75 bp paired-end reads.

#### OxBSSeq

DNA conversion and library preparation were performed using the CEGX TrueMethyl-Seq Whole Genome kit (Cambridge Epigenetix, Cambridge, UK) and after its acquisition from Tecan, the Ultralow Methyl-Seq with TrueMethyl OxBS Module (#0541 and #9513) following the manufacturer’s instructions. Briefly, the DNA was sheared to 800 bp using Covaris sonicator and treated with the oxidation agent and bisulfite following the manufacturer’s protocol. Libraries were sequenced on Illumina NextSeq 500 to yield 75 bp paired-end reads. The efficiency of the DNA oxidation and conversion was assessed by interrogating the spike-in Digestion Control and Sequencing Control using the dockerized custom pipeline bsExpress from CEGX (https://bitbucket.org/cegx-bfx/cegx_bsexpress) and fell within the expected ranges (≥90% for hmC conversion in the OxBs reaction, <10% for hmC conversion in the BS reaction (hmC/BS over-conversion error rate), <5% for mC conversion in both the OxBs and BS reactions (over-conversion error rate)).

#### ChIPSeq

Chromatin immunoprecipitation was performed using the Low Cell ChIPSeq kit (#53048, Active Motif) following the manufacturer’s instructions. Libraries were prepared using the supplied library prep reagents. The samples were sequenced on Illumina NextSeq 500 to yield 75 bp paired-end reads.

### Bioinformatic data analysis

Most data analysis was done in the R/Bioconductor environment (Huber et al., 2015) in RStudio (https://www.R-project.org/, http://www.rstudio.com/). For general processing, data exploration, and visualization, we used the tidyverse array of packages, in particular ggplot and dplyr (Wickham et al., 2019).

#### RNASeq

RNASeq reads were aligned using STAR (Dobin et al., 2013) and genome assemblies from UCSC. In addition to default STAR parameters, we used the following for paired-end data (--outFilterMismatchNmax 999 --alignMatesGapMax 1000000 -- outFilterScoreMinOverLread 0 --outFilterMatchNminOverLread 0 --outFilterMatchNmin 60 -- outFilterMismatchNoverLmax 0.05). Aligned reads were converted to bigwigs for visualization in IGV (Robinson et al., 2011) using deepTools (Ramírez et al., 2016). UCSC gene model annotations for whole genes were downloaded using the UCSC Table Browser tool. Transcript level quantifications was performed using Salmon (v1.1.0) (Patro et al., 2017) and imported for differential expression analysis with tximport (v1.10.1) (Soneson et al., 2015) and DESeq2 (v1.22.2) (Love et al., 2014). For up- and down-regulated genes, we selected for a log2 fold change of 2 and p-adjusted value of 0.05. GO analysis was performed using GOrilla (http://cbl-gorilla.cs.technion.ac.il/) (Eden et al., 2009) or DAVID (https://david.ncifcrf.gov) (Huang et al., 2009).

#### ATACSeq

Reads were processed with trim_galore (Martin, 2011) with parameters ‘--stringency 3 --fastqc --paired’. Trimmed reads were mapped to mm10 using bowtie2 (version 2.1.0) (Langmead and Salzberg, 2012) with parameters ‘-X 2000 --no-mixed --no-discordant’. Duplicates were removed using samtools (Li et al., 2009). Reads were normalized to RPKM using deepTools bamCompare module, ignoring chrX, chrY, chrM and filtering reads for minimum mapping quality of 30. Metagene and heatmap profiles were generated using deepTools modules computeMatrix, plotProfile, and plotHeatmap. Unique fragments under 100 nt were used to call peaks with macs2 (Zhang et al., 2008) with parameters ‘--nomodel -q 0.01 --call-summits’. Broad peaks were called with macs2 as well with parameters ‘--nomodel -f BAM --keep-dup all --broad -g mm -B -q 0.01’. The peaks were filtered to remove chrY and chrM peaks and peaks that overlap with the mm10 blacklist from ENCODE (ENCODE Project Consortium, 2012). DiffBind (v2.10.0) (Ross-Innes et al., 2012) was used to identify differentially accessible chromatin regions using the DESeq2 method. Peaks were selected based on fourfold difference and q-value of 0.05. Differential motif enrichment was performed using chromVAR (v1.4.1) (Schep et al., 2017).

#### OxBSSeq

Reads were processed with trim_galore with parameters ‘--stringency 3 --fastqc --paired --clip_R1 5 --clip_R2 10’. Trimmed reads were mapped to mm10 using bismark (Krueger and Andrews, 2011) with parameters ‘--bowtie2 -p 4 --multicore 4’ (v0.20.0). Duplicates were removed using deduplicate_bismark. The following tools from methpipe (v3.4.2) (Song et al., 2013) were used for downstream statistical estimation of the methylation and hydroxymethylation levels. First, bismark aligned reads were converted to the custom.mr format and sorted with default parameters. Methylation calls were extracted with methcounts with default parameters. Methylation and hydroxymethylation levels were estimated using the mlml tool with default parameters. Genome browser files were generated with bedGraphToBigWig. UMRs and low methylated regions (LMRs) were identified using MethylSeekR (v1.22.0) (Burger et al., 2013) with m = 0.5% and 5% FDR. DMVs were identified as UMRs ≥ 5 kb with mean.meth ≤ 15 and regions within 1 kb of each other were merged (bedtools merge -d 1000). mm10 CpG island annotations were downloaded from the UCSC table browser. Unique large DMVs were selected by excluding regions under 15 kb. The regions were annotated using HOMER (Heinz et al., 2010). DMR analysis was performed using methylpy (v1.3.4) (Schultz et al., 2015) with default parameters.

#### ChIPSeq

Reads were processed with trim_galore with parameters ‘--stringency 3 --fastqc --paired’. Trimmed reads were mapped to mm10 using bowtie2 (v2.1.0) with default parameters. Duplicates were removed using samtools. QC was performed with ChIPQC (Carroll et al., 2014) and that estimated fragment size was used for broad peak calling with macs2 (--nomodel -f BAM --keep-dup all --broad -g mm -B -q 0.01).

#### Antibody list

**Table:** 

Antigen	Species	Vendor	Cat #	RRID	Dilution
Itpr1* Clone ID: S24-18	Mouse	Origene	TA326547		FACS 1:500IF 1:500
Itpr1* S24-18	Mouse	Abcam	ab190239		FACS 1:500IF 1:500
Calb1	Rabbit	Immunostar	24427	AB_572222	IF 1:500
GFP	Chicken	Abcam	ab13970		IF 1:500
H3K27me3	Rabbit	Active Motif	39155	AB_2561020	ChIP 4 µL
H3K4me3	Rabbit	Active Motif	39159	AB_2615077	ChIP 4 µL
*Same clone, discontinued at Abcam					

## Data Availability

Pcp2TetTKO mice will be made available with a Material Transfer Agreement (MTA). Sequencing data is available on NCBI Gene Expression Omnibus under accession number GSE166423. Code for data analysis is available at https://github.com/estoyanova/EStoyanova_eLife_2021 copy archived at https://archive.softwareheritage.org/swh:1:rev:4c2fa6c102e4f868fc91e0dcb0b8c7155b8f8712. The following dataset was generated: StoyanovaE RiadM RaoA HeintzN 20215-hydroxymethylcytosine is required for terminal differentiation of Purkinje neuronsNCBI Gene Expression OmnibusGSE166423

## Additional files

Supplementary file 1. Developmental differentially expressed genes between P0 and adult Purkinje cells.

Supplementary file 2. DNA methylation valleys (DMVs) identified in adult Purkinje neurons.

Supplementary file 3. Differentially expressed genes in the adult triple Tet proteins knockout.

Transparent reporting form
