# Supplementary material for: 5-Hydroxymethylcytosine-mediated active demethylation is required for mammalian neuronal differentiation and function
Source: eLife. 2021 Dec 17;10:e66973. doi: 10.7554/eLife.66973 (PMC8683082; doi:10.7554/eLife.66973)
Supplement: Peer review history

# Editor report: Editor's evaluation

**DOI:** 10.7554/eLife.66973.sa0

**Authors:** Anne E West

**Affiliations:** Duke University School of Medicine United States

This study comprehensively characterizes the regulation of chromatin in developing Purkinje neurons of the cerebellum and demonstrates a role for the Tet family of oxidases in controlling temporal changes in gene transcription during differentiation via active DNA demethylation.

# Decision letter

**DOI:** 10.7554/eLife.66973.sa1

**Affiliations:** Duke University School of Medicine United States

In the interests of transparency, eLife publishes the most substantive revision requests and the accompanying author responses.

[Editor’s note: review comments and author responses relating to electrophysiology data, which were included in an earlier version, have been omitted.]

Congratulations, we are pleased to inform you that your article, "5-hydroxymethylcytosine mediated active demethylation is required for neuronal differentiation and function", has been accepted for publication in eLife.

Thank you for submitting your work entitled "5-hydroxymethylcytosine mediated active demethylation is required for neuronal differentiation and function" for consideration by *eLife*. Your article has been reviewed by 3 peer reviewers, one of whom is a member of our Board of Reviewing Editors, and the evaluation has been overseen by a Reviewing Editor and a Senior Editor. The reviewers have opted to remain anonymous.

*Reviewer #1 (Recommendations for the authors):*

In this manuscript the authors carry out epigenomic profiling purified Purkinje cells across key stages of cerebellar development to study the regulation and function of 5hmC. The authors have previously published a method for isolation of FACS-based nuclear sorting for these cells and here they use it to isolate from P0, P7, and adult cerebellum with high quality RNA, mC/hmC, ATAC and ChIP-seq data at each time point in biological replicate. The chromatin data are correlated against developmental changes in gene expression and suggest broad mechanisms of chromatin control that are, for the most part, in line with previous studies of other cell types. The authors are particularly interested in regions of the genome that show loss of mCG (and hmCG) over developmental time and are near genes that show enhanced expression in adult PCs. They test the requirement for the Tets in the developmental demethylation of these regions by knocking out all three Tets in a PC specific manner at P7, then running RNA/mCG/hmCG sequencing in adult to compare between cTKO and control. 

PCs are a biologically important class of neurons and they are a very different cell type from others that have been developmentally studied. For these reasons the descriptive chromatin data gathered here are of use to the community. However, the analyses performed on the data as presented here are somewhat underdeveloped. I

*Reviewer #2 (Recommendations for the authors):*

TET dioxygenases (TET1, TET2, TET3) mediate the conversion of 5-methylcytosine (5mC) to 5-hydroxymethylcytosine (5hmC), which may lead to additional forms of DNA modifications and ultimately DNA demethylation. In dividing cells, the maintenance DNA methyltransferase recognizes poorly of 5hmC, which leads to passive DNA demethylation through replacing the 5hmC sites with unmethylated cytosine after rounds of DNA replication. However, whether 5hmC undergoes demethylation in non-dividing cells, for example through TDG-BER dependent active DNA demethylation, and how it may impact gene transcription and cellular properties remain elusive. To answer these questions will particularly expand our current understanding of TET catalyzed methylcytosine oxidation in the brain, the organ that has not only the highest levels of 5hmC, but also thousands of millions of non-dividing neurons that remain plastic throughout life.

Purkinje neurons in cerebellum subject to orchestrated postnatal migration, arborization, and synaptogenesis after their final cell division at mid-gestation stage. The study focuses on this neuron type and generates single base resolution 5mC and 5hmC mapping at three developmental stages (P0, P7, young adult). In addition, it profiles the Purkinje neuron specific transcriptiome (nuclei RNAseq), epigenome (H3K4me3, H3K27me3), and chromatin accessibility (ATACseq). The study therefore displays novel modes of epigenomic alterations that are associated with transcription regulation, which also confirms DNA methylation turnovers during Purkinje neuron post-mitotic maturation. Furthermore, the study generates a conditional knockout mouse line with all three TET enzymes selectively deleted in Purkinje neurons. The deficiency of TET enzymes not only impedes the DNA methylation changes during Purkinje cell postnatal maturation, but also alters neuronal excitability that increases susceptibility to an excitotoxic drug. Therefore, the study demonstrates a requirement of dynamic 5mC/5hmC changes during cerebellum Purkinje neuron post-mitotic maturation and their implication in neuronal function.

The study has successfully addressed a few technical challenges, such as the low abundancy of Purkinje neurons, to profile 5mC and 5hmC separately, and to generate a cell type specific triple TET knockout mouse line. The data are of good quality (e.g. Figure S1). Overall, the conclusions are supported by the data. Epigenetic modifications have been known to play cell type specific functions. However, the majority of current research is limited by the heterogeneity of tissue samples that lack a cell type specific resolution. To study DNA modifications in a defined neuron type, as shown in this manuscript, provides valuable novel insights (e.g. as mentioned in the Discussion, the changes are specific to Purkinje cells, but not observed in cerebellum granule neurons). In addition to sequencing 5mC and 5hmC side by side, the study also analyzes CG and CH regions separately. The study recognizes unique patterns of 5mC, 5hmC, CG and CH changes, respectively, which suggest the complexity of DNA modification in the brain. The set of genes that are associated with DNA methylation valley dynamics are particularly interesting. The genomic datasets and the mouse line derived from this study will also serve as a good resource for related work.

Through RNAseq, less than 2000 genes are found to be differentially expressed during Purkinje neuron maturation. The study therefore spends major efforts on analyzing the epigenetic changes at these genes. However, it is not sufficiently clear of the epigenetic status of the vast majority of genome beyond the differentially expressed genes, even though some indicative findings are revealed at regions with "gain" or "loss" of chromatin accessibility (Figure 2K-N). To assess this, it will be helpful to further characterize intergenic regions in the future, for example through genomic profiling of enhancer markers H3K4me1 and H3K27ac.

An enhancer marker ChIPseq is preferred, but not required. Below are some suggested revision points.

1. It will be informative to demonstrate the transcription levels of the three TET family members during Purkinje neuron maturation. This can be obtained from the RNAseq data that the authors have generated.

2. The transcriptome and epigenome differential lists should be provided in supplements.

3. It is unclear if both males and females are included in the genomic assays and animal work.

4. Though the number of cells utilized in each genomic assay is stated in methods, it will be helpful to describe the corresponding number of animals.

[Editors’ note: the authors submitted for reconsideration following the decision after peer review. What follows is the decision letter after the first round of review.]

Thank you for resubmitting your work entitled "5-hydroxymethylcytosine mediated active demethylation is required for neuronal differentiation and function" for further consideration by *eLife*. Your revised article has been evaluated by Catherine Dulac (Senior Editor) and a Reviewing Editor.

The manuscript has been improved but there are some remaining issues that need to be addressed, as outlined below:

*Reviewer #1 (Recommendations for the authors):*

In the first round of review I raised two main concerns about the molecular data – the novelty of the findings and the depth of the analysis linking Tet-mediated DNA demethylation with PC maturation. One of the other reviewers raised concerns about the physiology of the Tet cKO mice.

1. Novelty of the findings

The first concern was the degree of novelty in showing Tet-mediated DNA demethylation in post-mitotic neurons. My concern arose from the language used by the authors in their manuscript. In the abstract of the revised manuscript they state, "is it not known whether 5hmC is required for terminal differentiation or if it can serve as an intermediate in active DNA demethylation in postmitotic neurons." This assertion does not jibe with my reading of the literature. My concern was amplified when I read the sentence at line 68 in the introduction that says, "Tet mediated replication dependent passive demethylation is thought to be common in many dividing cell types, including progenitor cells in the developing forebrain (Rudenko et al. 2013), hippocampus (Guo et al. 2011) and cerebellum (Zhu et al. 2016)." This is an inaccurate statement regarding these papers from the literature.

Rudenko et al. studied DNA methylation in adult brains of a Tet1 knockout mouse and found hypermethylation of activity-regulated gene promoters in the knockout compared with wildtype. I appreciate that because this was a germline knockout that this study does not rule out the possibility that the increases in methylation occurred in dividing progenitor cells and were maintained. However, the authors of the current manuscript are over-reaching to conclude that the changes in methylation seen in the Rudenko study were indeed the result of passive demethylation in progenitors, given that this was not tested in the study. The Guo and Zhu studies knocked down Tets in postmitotic neurons. In the Guo 2011 study the authors injected shRNA-bearing AAVs targeting Tet1 in adult dentate gyrus. In the Zhu 2016 study the authors lentivirally infected primary CGN cultures with shRNAs targeting Tets 1 and 3. Both studies showed increases in DNA methylation in knockdown compared with wildtype neurons. In the Guo study this was in the context of seizure-induced demethylation of the Bdnf and Fgf1b genes, and the demethylation was blocked by Tet1 KD. Although there are a few progenitors in the adult dentate gyrus, it is not reasonable to attribute the methylation effects to only these few cells if that is what the authors mean by saying this study was in progenitors. The authors of the Guo paper concluded that Tet1 acted to actively demethylate DNA in postmitotic hippocampal neurons, and their data support that conclusion. The Zhu 2016 used primary CGNs in culture, which have been shown by many groups (using the specific protocol derived from the Hatten lab) to be fully post-mitotic within the first 24 hours after plating. shRNA-mediated knockdown occurs on the order of days (cells were studied at 3DIV). Therefore, the assumption of Guo 2016 in their conclusions that the Tets are acting to mediate these changes in post-mitotic neurons is justified.

Later (line 274) the manuscript references Lio 2016 to cite that prior studies have established that the activation of enhancers is accompanied by increased accessibility and loss of DNA methylation. At line 291 the authors suggest that the DNA demethylation they find in PCs is novel because "in this case…DNA demethylation does not require cell division." I agree it is true the PC data shows postmitotic demethylation, however it was also shown in PMID 32589877. In that study the authors purified nuclei of SST+ and VIP+ neurons from the mouse cortex during development. These experiments were done during comparing interneuron nuclei isolated at P7 with those from P13, both stages at which these neurons are already postmitotic.

These discrepancies in the manuscript between the authors' assertions of what these studies showed and the published data need to be corrected to be accurate.

It was because the authors suggested the novelty of their study as the first to show Tet-mediated active DNA demethylation in postmitotic neurons that I questioned that novelty. As the literature review above shows, this manuscript is not the first to show Tet-mediated active DNA demethylation in postmitotic neurons.

However, this manuscript does do a particularly nice job of it. I think the novelty of the study lies in linking the molecular characterization of PC chromatin regulation during development with the biology of this cell population. With this in mind, I agree with the authors that the body of the data and the analysis do make a significant new addition to the literature that will be of broad interest.

2. Depth of the analysis linking Tet-mediated DNA demethylation with PC maturation

In this revision the authors have significantly expanding and clarified the section on the molecular sequencing from Tet_1/2_/3 cKO mice, strengthening the impact of the data. However, even though this is a revised manuscript there are some important omissions and errors in this section that undermine the power of the data to support the model.

– Line 193, 5hmc continues to increase over highly expressed genes – where is that evidence?

– Line 202 "Furthermore, 5hmCG accumulated over the genes bodies early in development is not removed (Figure 2C)," actually what this figure shows is that for the downregulated genes there is an increase in 5hmCG over the gene bodies between P7 and adult, so it is not only not lost, it is gained. It looks no different for the downregulated genes than the upregulated genes.

– Where is the data that goes with line 372 – growth of the DMV preceded by 5hmC and starts at 5' end

– Line 376 -The text that follows this (to line 381) is entirely speculative and does not belong in the methods.

– Figure 4S2 E-G are not cited in the text.

– Line 496 – the authors state they have not seen demethylation in CGNs with no attribution and no data. Also, this contrasts with the Zhu 2016 paper so should be discussed in comparison.

Furthermore, one of the important parts of this section regards the relationship of the chromatin changes in the Tet cKO to the gene expression effects and this part would benefit from more discussion. For example, one of the best example genes for Tet mediated demethylation the authors show it Iptr and yet they specifically say this epigenomic change does not influence Iptr expression (which is key since this is the gene product used for PC isolation). Also, it is surprising that in the Tet_1/2_/3 cKO mice, expression of genes encoding synaptic proteins and differentiation markers are the major class elevated, not repressed. Might this suggest that the Tets actually slow differentiation rather than promoting it as the text seems to imply? Either would be interesting, but the data shown needs to be reconciled with the statement (line 106) that "maturation in Purkinje Cells … require continued oxidation of 5mC to 5hmC".

# Reply: Author response

**DOI:** 10.7554/eLife.66973.sa2

Reviewer #1 (Recommendations for the authors):

In this manuscript the authors carry out epigenomic profiling purified Purkinje cells across key stages of cerebellar development to study the regulation and function of 5hmC. The authors have previously published a method for isolation of FACS-based nuclear sorting for these cells and here they use it to isolate from P0, P7, and adult cerebellum with high quality RNA, mC/hmC, ATAC and ChIP-seq data at each time point in biological replicate. The chromatin data are correlated against developmental changes in gene expression and suggest broad mechanisms of chromatin control that are, for the most part, in line with previous studies of other cell types. The authors are particularly interested in regions of the genome that show loss of mCG (and hmCG) over developmental time and are near genes that show enhanced expression in adult PCs. They test the requirement for the Tets in the developmental demethylation of these regions by knocking out all three Tets in a PC specific manner at P7, then running RNA/mCG/hmCG sequencing in adult to compare between cTKO and control. Finally, the authors assess the functional importance of Tet-dependent gene regulation by recording from cTKO and control PCs and assessing behavioral features of PC function (rotarod and harmaline-induced tremor).

PCs are a biologically important class of neurons and they are a very different cell type from others that have been developmentally studied. For these reasons the descriptive chromatin data gathered here are of use to the community. However, the analyses performed on the data as presented here are somewhat underdeveloped. It is not clear to me that the evidence for developmental demethylation is novel and the data in Figure 4 (which could be very powerful) are not strongly deployed to argue that Tets are key in the demethylation process.

Reviewers 1 and 2 both appreciate that the data we present for transcription, DNA methylation and hydroxymethylation, chromatin accessibility, H4K4me3 and H3K27me3 histone marks are high quality and valuable for the community. We agree with this assessment, and we present them both to support our specific interests and as a resource for further analysis. With respect to our analysis being characterized as “somewhat underdeveloped”, these data are so massive that a complete study of all of their features will require many additional sets of analyses and follow up studies. There is little doubt in our minds that the data will be embraced by the community and additional biological inferences will emerge from them. Although we could populate several more figures with further analysis of these data, we have chosen to focus on only those findings supporting the most novel and important conclusions relative to the direct roles of 5hmC in differentiation.

Novelty. It has been demonstrated that the distribution of 5hmC in neuronal genomes is cell type specific and correlates with active gene expression, and that germline deletions of Tet oxidases disrupt development of the brain. Reviewer 1 is familiar with this “developmental role” of 5hmC covering the course of neuronal development – including proliferative phases of neuronal progenitor expansion where 5hmC is required for replication dependent DNA demethylation and chromatin remodeling. Replication dependent DNA demethylation is a fundamental feature of DNA methylation refinement that occurs in many cell types. However, the term “developmental demethylation” does not distinguish between the role of 5hmC mediated, replication dependent passive demethylation in progenitors and the proposed, but not proven, role of 5hmC mediated active demethylation in the absence of cell division (Ito et al., 2011). Our study was designed to arrest 5hmC formation in postmitotic differentiating PCs to address specifically this issue. The design of the study, the use of new cell type specific triple Tet1, Tet2, Tet3 triple knockout lines that activate well after the final division of PC progenitors, and the results obtained are novel and of high interest.

Figure 4. To address the remaining comments of Reviewer 1, we have edited substantially the section of the manuscript subtitled “Loss of 5mC and 5hmC in active genes during PC differentiation” presenting the data documenting loss of both 5mC and 5hmC in a specific class of highly expressed genes during PC differentiation. We believe that these new edits present the data in a concise yet very clear manner that is accessible to even a general reader. In doing so, they also augment the following section “Loss of 5mC and 5hmC in putative regulatory sites during PC differentiation”. We think this improved presentation of the data in Figure 3 establishes for the reader both that DNA demethylation occurs as PC differentiation proceeds, and that 5hmC accumulation is present in the P7 data at these sites prior to the evident loss of both 5mC and 5hmC in the adult. These new edits provide an improved introduction to Figure 4, and we believe they will help the reader understand more readily the molecular events that are disrupted as a consequence of loss of 5hmC.

Of course, to provide proof that loss of DNA methylation over the PC DMVs is dependent on 5hmC required disruption of continued 5hmC production in the Pcp2TetTKO mice. Figure 4 presents first the basic characteristics of the Pcp2TetTKO lines, establishing that their survival and body weight is normal (panels B,C), and that no gross motor phenotype is evident in rotarod performance measures (panel D). The remaining data in Figure 4 are meant to address directly the molecular events that occur in Pcp2TetTKO PCs and the requirement for 5hmC in the DNA demethylation described in Figure 3.

The description of the molecular events that occur in PCP2TetTKO PCs has been extensively lengthened to clarify each point revealed by the data. We have also changed panel G to box and whisker plots so that the reader can more easily evaluate the alterations in methylation over ATAC peaks (putative regulatory sites) that both increase in adult PCs and are associated with genes whose expression changes in the Pcp2TetTKO PCs. We hope that the additional detailed discussion of the data and our conclusions will address the comment that this section of the manuscript is “somewhat underdeveloped”.

Reviewer #2 (Recommendations for the authors):

[…] Through RNAseq, less than 2000 genes are found to be differentially expressed during Purkinje neuron maturation. The study therefore spends major efforts on analyzing the epigenetic changes at these genes. However, it is not sufficiently clear of the epigenetic status of the vast majority of genome beyond the differentially expressed genes, even though some indicative findings are revealed at regions with "gain" or "loss" of chromatin accessibility (Figure 2K-N). To assess this, it will be helpful to further characterize intergenic regions in the future, for example through genomic profiling of enhancer markers H3K4me1 and H3K27ac.

An enhancer marker ChIPseq is preferred, but not required. Below are some suggested revision points.

1. It will be informative to demonstrate the transcription levels of the three TET family members during Purkinje neuron maturation. This can be obtained from the RNAseq data that the authors have generated.

2. The transcriptome and epigenome differential lists should be provided in supplements.

3. It is unclear if both males and females are included in the genomic assays and animal work.

4. Though the number of cells utilized in each genomic assay is stated in methods, it will be helpful to describe the corresponding number of animals.

We thank Reviewer 2 for a general appreciation of our study, and we believe the new edits to the manuscript will strengthen the already positive description of our work “To study DNA modifications in a defined neuron type, as shown in this manuscript, provides valuable novel insights (e.g. as mentioned in the Discussion, the changes are specific to Purkinje cells, but not observed in cerebellum granule neurons). In addition to sequencing 5mC and 5hmC side by side, the study also analyzes CG and CH regions separately. The study recognizes unique patterns of 5mC, 5hmC, CG and CH changes, respectively, which suggest the complexity of DNA modification in the brain. The set of genes that are associated with DNA methylation valley dynamics are particularly interesting. The genomic datasets and the mouse line derived from this study will also serve as a good resource for related work.” We agree with Reviewer 2 that it “will be helpful to further characterize intergenic regions in the future, for example through genomic profiling of enhancer markers H3K4me1 and H3K27ac.” While these and many other types of experiments are certainly of interest, we view these as future studies that will continue to reveal interesting details that occur during the prolonged and complex differentiation programs characteristic of Purkinje cells and other principal neurons.

We have addressed in the revised manuscript all of the additional, detailed comments of Reviewer 2 and included additional data on the levels of expression of the Tet enzymes as PCs mature into Figure 4 Supplement 1A. Of course, the final transcriptome and epigenome lists will be provided with final manuscript. We believe these changes have improved the manuscript and thank Reviewer 2 for highlighting these issues.

[Editors’ note: the authors submitted for reconsideration following the decision after peer review. What follows is the decision letter after the first round of review.]

Reviewer #1 (Recommendations for the authors):

In the first round of review I raised two main concerns about the molecular data – the novelty of the findings and the depth of the analysis linking Tet-mediated DNA demethylation with PC maturation.

1. Novelty of the findings

The first concern was the degree of novelty in showing Tet-mediated DNA demethylation in post-mitotic neurons. My concern arose from the language used by the authors in their manuscript. In the abstract of the revised manuscript they state, "is it not known whether 5hmC is required for terminal differentiation or if it can serve as an intermediate in active DNA demethylation in postmitotic neurons." This assertion does not jibe with my reading of the literature. My concern was amplified when I read the sentence at line 68 in the introduction that says, "Tet mediated replication dependent passive demethylation is thought to be common in many dividing cell types, including progenitor cells in the developing forebrain (Rudenko et al. 2013), hippocampus (Guo et al. 2011) and cerebellum (Zhu et al. 2016)." This is an inaccurate statement regarding these papers from the literature.

We agree with Reviewer 1 that our interpretation of these studies is rather strict. In the revised manuscript we have expanded this section to delineate the findings in each of these studies. We have added a reference n line 94 for a review of studies of 5hmC and passive demethylation in neural progenitors (MacArthur and Dawlaty, 2021). We have also included, in accord with first Reviewer’s comments, a new paragraph that explicitly states the findings of previous studies of demethylation including the mentioned references. We state that these studies provide evidence that is consistent with the hypothesis that there is active DNA demethylation in post-mitotic neurons. However, in our judgement they do not constitute definitive proof of active DNA demethylation in vivo. Consequently, this paragraph concludes with the sentence “Despite these observations, our knowledge of the roles of continued 5hmC accumulation in active DNA demethylation and postmitotic differentiation remain rudimentary. “

Rudenko et al. studied DNA methylation in adult brains of a Tet1 knockout mouse and found hypermethylation of activity-regulated gene promoters in the knockout compared with wildtype. I appreciate that because this was a germline knockout that this study does not rule out the possibility that the increases in methylation occurred in dividing progenitor cells and were maintained. However, the authors of the current manuscript are over-reaching to conclude that the changes in methylation seen in the Rudenko study were indeed the result of passive demethylation in progenitors, given that this was not tested in the study.

We agree with the Reviewer 1 that it is possible that the “hypermethylation” observed in this study results from demethylation in post-mitotic neurons. However, this is a germline KO so these changes could have occurred by passive demethylation in dividing progenitors during development. Furthermore, the assay used to assess these changes is bisulfite sequencing – it does not distinguish between 5mCG and 5hmCG. We agree with Reviewer 1 that no data are presented to directly address this issue, so we have edited the text to discuss this possibility in the revision.

The Guo and Zhu studies knocked down Tets in postmitotic neurons. In the Guo 2011 study the authors injected shRNA-bearing AAVs targeting Tet1 in adult dentate gyrus. In the Zhu 2016 study the authors lentivirally infected primary CGN cultures with shRNAs targeting Tets 1 and 3. Both studies showed increases in DNA methylation in knockdown compared with wildtype neurons. In the Guo study this was in the context of seizure-induced demethylation of the Bdnf and Fgf1b genes, and the demethylation was blocked by Tet1 KD. Although there are a few progenitors in the adult dentate gyrus, it is not reasonable to attribute the methylation effects to only these few cells if that is what the authors mean by saying this study was in progenitors. The authors of the Guo paper concluded that Tet1 acted to actively demethylate DNA in postmitotic hippocampal neurons, and their data support that conclusion.

We agree that it is possible that the increase in “methylation” observed by Guo et al. could arise by the inability to demethylate these sites. However, the assay here was again bisulfite sequencing that cannot distinguish 5mC and 5hmC. Furthermore, the protocol was to use electro convulsive stimulation to induce these changes. Since many studies have documented a strong relationship between 5hmC accumulation and transcription, since ECS causes a large increase in transcription from these promoters, and since both the Tet1 and Apobec1 knock downs leave at least Tet 2 and Tet3 activity intact, it is equally likely that the increased “methylation” seen in the KD is 5hmC rather than 5mC. Again, there are no data presented in the study to distinguish between these two possibilities. We have edited the text to clarify this issue and discuss the possibility that demethylation could occur.

The Zhu 2016 used primary CGNs in culture, which have been shown by many groups (using the specific protocol derived from the Hatten lab) to be fully post-mitotic within the first 24 hours after plating. shRNA-mediated knockdown occurs on the order of days (cells were studied at 3DIV). Therefore, the assumption of Guo 2016 in their conclusions that the Tets are acting to mediate these changes in post-mitotic neurons is justified.

The CGC cultures were prepared at P7, the time of maximal GC division in the EGL (Fujita et al. 1966, Fujita, 1967). As demonstrated by the Hatten lab (Gao et al., 1991), cell division in cultured granule cells depends on the plating density. There are no data in the paper to rule out the possibility that a subpopulation of the plated GCs divide for a day or two after being cultured. Furthermore, this paper is dedicated to showing that 5hmC is required for gene expression and does not mention demethylation.

The discussion of Figure 6 states that “5hmC levels decreased dramatically in transfected cells, while 5mC levels increased slightly. There are no statistics for this slight increase in 5mC. The word demethylation occurs only in the titles of papers cited in the References. In my opinion, this “slight increase” can’t be cited as proof of demethylation. However, we agree that this is a nice demonstration of the role of 5hmC in differentiation of granule cells and have edited the manuscript accordingly.

Later (line 274) the manuscript references Lio 2016 to cite that prior studies have established that the activation of enhancers is accompanied by increased accessibility and loss of DNA methylation. At line 291 the authors suggest that the DNA demethylation they find in PCs is novel because "in this case…DNA demethylation does not require cell division." I agree it is true the PC data shows postmitotic demethylation, however it was also shown in PMID 32589877. In that study the authors purified nuclei of SST+ and VIP+ neurons from the mouse cortex during development. These experiments were done during comparing interneuron nuclei isolated at P7 with those from P13, both stages at which these neurons are already postmitotic.

Although we thank Reviewer 1 for stimulating us to more accurately discuss the three papers cited above, we do not agree with the statement that postmitotic demethylation “was also shown in PMID 32589877”. The paper in this citation “An Activity-Mediated Transition in Transcription in Early Postnatal Neurons” (Stroud et al., Neuron, 2020) is a very nice study of enhancer activation and decommissioning in the developing brain. In the relevant section concerning the role of DNA methylation, the authors use Sst and Vip Cre drivers to knockout Dnmt3a in these interneuron types. The result is that the increase in methylation that occurs normally at these sites does not occur in the KO. The fact that there is less methylation in the KO reflects the failure of DNMT3A to methylate these sites, not DNA demethylation. The word “demethylation” does not appear in the paper. While the Stroud et al. study is very well done, it is not directly relevant and we have not cited it.

These discrepancies in the manuscript between the authors' assertions of what these studies showed and the published data need to be corrected to be accurate.

It was because the authors suggested the novelty of their study as the first to show Tet-mediated active DNA demethylation in postmitotic neurons that I questioned that novelty. As the literature review above shows, this manuscript is not the first to show Tet-mediated active DNA demethylation in postmitotic neurons.

However, this manuscript does do a particularly nice job of it. I think the novelty of the study lies in linking the molecular characterization of PC chromatin regulation during development with the biology of this cell population. With this in mind, I agree with the authors that the body of the data and the analysis do make a significant new addition to the literature that will be of broad interest.

To address these comments, we have edited the manuscript to state accurately what has been established in the literature. My strict technical interpretation of the cited papers is that the caveats discussed above prevent a definitive statement that DNA demethylation occurs in postmitotic neurons. This is the reason we went to great lengths to obtain precise data using the most advanced technology (oxBS) that can distinguish 5mC from 5hmC, and why we constructed PC specific triple knockout lines that are activated at P7 to complete this study. we understand, however, that we have been too strident and we have tried to edit the manuscript according to the comments of Reviewer 1.

2. Depth of the analysis linking Tet-mediated DNA demethylation with PC maturation

In this revision the authors have significantly expanding and clarified the section on the molecular sequencing from Tet_1/2_/3 cKO mice, strengthening the impact of the data. However, even though this is a revised manuscript there are some important omissions and errors in this section that undermine the power of the data to support the model.

– Line 193, 5hmc continues to increase over highly expressed genes – where is that evidence?

This is shown in Figure 2S1E. As a result of an editorial error, we did not include the figure citation in the manuscript, and the text has now been edited to correct this error.

– Line 202 "Furthermore, 5hmCG accumulated over the genes bodies early in development is not removed (Figure 2C)," actually what this figure shows is that for the downregulated genes there is an increase in 5hmCG over the gene bodies between P7 and adult, so it is not only not lost, it is gained. It looks no different for the downregulated genes than the upregulated genes.

We agree with the reviewer, and we have edited the manuscript to state that 5hmC continues to increase over these genes.

– Where is the data that goes with line 372 – growth of the DMV preceded by 5hmC and starts at 5' end

These two points are illustrated in (Figure 4H, I). In panel 4H, one can clearly see in the IGV panel that the level of 5hmC at significantly greater at P7 than in the adult over nearly the entire gene body for GPR63, and that this is also the case at the 5’end of Grid2. This is more difficult to appreciate for Grid2 because it is such a large gene and the demethylation occurs only over the first 50-100 kb of the gene. The metagene plots in Figure 4I also indicate that for this whole class of genes, the 5hmC levels are high at P7 and much less in the adult indicating that 5hmC accumulated during development is lost as the cells differentiate.

– Line 376 -The text that follows this (to line 381) is entirely speculative and does not belong in the methods.

Quote in question “*These data revealed a large variation in the length of the DMV that does not form in the Pcp2TetTKO PCs. We believe this reflects a complex relationship between the timing at which transcription is initiated, the rate of transcription over the entire gene body, the local activity of Tet oxidase within the gene as differentiation proceeds, and the timing of Tet oxidase loss from each cell following recombination at the Tet1, Tet2, and Tet3 loci.* “

We agree with Reviewer 1 that this is speculation. The fact that the sentence starts with “We believe” clearly indicates that this is our conjecture and not a verified result. We see no reason why we can’t let the reader evaluate this idea for themselves, and we would like to leave this in the Results as it is stated.

– Figure 4S2 E-G are not cited in the text.

We made an unfortunate typo while referring to the supplemental figures. Figures 4S2 E-G were incorrectly referred to as 4S1 E-G. This has been corrected in the revision.

Furthermore, one of the important parts of this section regards the relationship of the chromatin changes in the Tet cKO to the gene expression effects and this part would benefit from more discussion. For example, one of the best example genes for Tet mediated demethylation the authors show it Iptr and yet they specifically say this epigenomic change does not influence Iptr expression (which is key since this is the gene product used for PC isolation). Also, it is surprising that in the Tet_1/2_/3 cKO mice, expression of genes encoding synaptic proteins and differentiation markers are the major class elevated, not repressed. Might this suggest that the Tets actually slow differentiation rather than promoting it as the text seems to imply? Either would be interesting, but the data shown needs to be reconciled with the statement (line 106) that "maturation in Purkinje Cells…..require continued oxidation of 5mC to 5hmC".

We appreciate this comment and we understand that in the simplest case one might interpret the findings as consistent with slowing differentiation. However, we don’t know whether late expressed genes in PCs may be expressed at higher levels during synaptogenesis than they are once they reach their steady state levels in the adult. We chose the word maturation to reflect this uncertainty. We have also edited the last sentence of this paragraph to state more precisely our conclusion “Taken together, our data demonstrate that active demethylation occurs in select genes in postmitotic neurons, and that 5hmC plays an essential role in refining the transcriptional and epigenetic status of Purkinje cells during the final stages of differentiation.”
